# Reformulating Reactivity Design for Data-Efficient Machine Learning

**DOI:** 10.1021/acscatal.3c02513

**Published:** 2023-10-06

**Authors:** Toby Lewis-Atwell, Daniel Beechey, Özgür Şimşek, Matthew N. Grayson

**Affiliations:** † Department of Chemistry, University of Bath, Claverton Down, Bath BA2 7AY, U.K.; ‡ Department of Computer Science, University of Bath, Claverton Down, Bath BA2 7AY, U.K.

**Keywords:** machine learning, activation barriers, catalyst design, organic synthesis, data efficiency

## Abstract

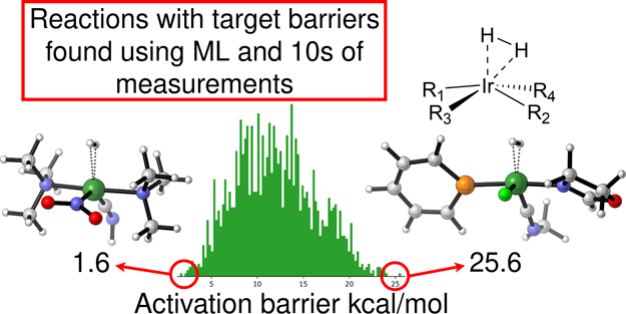

Machine learning (ML) can deliver rapid and accurate reaction barrier predictions for use in rational reactivity design. However, model training requires large data sets of typically thousands or tens of thousands of barriers that are very expensive to obtain computationally or experimentally. Furthermore, bespoke data sets are required for each region of interest in reaction space as models typically struggle to generalize. We have therefore reformulated the ML barrier prediction problem toward a much more data-efficient process: finding a reaction from a prespecified set with a desired target value. Our reformulation enables the rapid selection of reactions with purpose-specific activation barriers, for example, in the design of reactivity and selectivity in synthesis, catalyst design, toxicology, and covalent drug discovery, requiring just tens of accurately measured barriers. Importantly, our reformulation does not require generalization beyond the domain of the data set at hand, and we show excellent results for the highly toxicologically and synthetically relevant data sets of aza-Michael addition and transition-metal-catalyzed dihydrogen activation, typically requiring less than 20 accurately measured density functional theory (DFT) barriers. Even for incomplete data sets of E2 and S_N_2 reactions, with high numbers of missing barriers (74% and 56% respectively), our chosen ML search method still requires significantly fewer data points than the hundreds or thousands needed for more conventional uses of ML to predict activation barriers. Finally, we include a case study in which we use our process to guide the optimization of the dihydrogen activation catalyst. Our approach was able to identify a reaction within 1 kcal mol^–1^ of the target barrier by only having to run 12 DFT reaction barrier calculations, which illustrates the usage and real-world applicability of this reformulation for systems of high synthetic importance.

## 1 Introduction

The ability to determine the activation barriers of chemical reactions is highly desirable, allowing us to address interesting and important chemical problems. For example, understanding reaction kinetics^1,2^ or elucidating reaction mechanisms.^3−5^ Exposing this knowledge can greatly aid in the design of useful materials or new pharmaceutical compounds.^6−11^ Unfortunately, traditional methods of evaluating reaction activation barriers, quantum mechanical (QM) calculations or experiments, are very expensive. Therefore, demand for cheaper yet equally effective alternatives is high.^12−14^ Of particular recent interest is the use of machine learning (ML) models to predict activation barriers.^15^ The general ML approach to predict barriers is foundational: (1) obtain a data set of accurately measured activation barriers (either from experiment,^16^ or more commonly, quantum mechanical calculations^17−21^), (2) choose a suitable chemical representation, (3) train the ML model to minimize the regression loss between its outputs and the activation barriers of the training set. Typically, these data sets contain between thousands^19,20^ and tens of thousands^17,21^ of reaction barriers. As one illustration of the expense of creating such data sets, the quantum mechanical (QM) calculations for a set of E2 and S_N_2 reactions, crafted by von Rudorff et al., took approximately 2.8 million core hours to complete,^22^ and this data set provides the barriers for only 2720 symmetrically unique reactions.

Two recent studies have shown good results for predicting activation barriers in low data regimes. Friederich et al.^20^ used Gaussian process regression to predict the barriers of the dihydrogen activation reaction on derivatives of Vaska’s complex, calculated at the PBE-D3/def2-SVP level of theory. They were able to achieve a mean absolute error (MAE) in the barrier predictions below the chemical accuracy threshold of 1 kcal mol^–1^, using only 20% of the total data set size of 1947 reactions (approximately 390 reactions). Jorner et al.^16^ were also able to obtain an MAE below 1 kcal mol^–1^ when training a Gaussian process regression model on approximately 110 of a total of 443 experimentally derived nucleophilic aromatic substitution barriers. However, this did require one of the model’s input features to be the reaction barrier itself, calculated with density functional theory (DFT).

Despite some good results on small data sets, current ML approaches still suffer from another key limitation: They tend to show poor predictive performance on inputs lying outside the domain of their training data.^23−25^ For these methods to be widely adopted, they must show accurate predictions for all regions of interest in the reaction space. However, given the incomprehensible vastness of chemical space and the even larger number of possible reactions, the prospect of gathering enough accurate reference data to train ML models to predict arbitrary reaction barriers is infeasible.

Recently, some attention had been paid to addressing this infeasibility with transfer learning: Training a base model on a large initial data set containing a wide variety of different reactions and then fine-tuning on a small amount of data specific to a new reaction type. This approach has been reported to be useful for predicting the products of organic reactions,^26^ the geometries of the transition states of organic reactions,^27^ material properties,^28^ and activation barriers of Diels–Alder reactions.^29^ However, the true effects of transfer learning are still unclear. For instance, the base models for this type of transfer learning will still only be specific to a limited range of reaction classes, and transferring from them to new, very different reactions may not be effective or improve data efficiency.^30−32^

In this work, we contribute an alternative formulation for predicting reaction activation barriers in a highly data-efficient manner: identifying a reaction with a barrier close to a desired “target” value. Given the reaction class and desired activation barrier, we define a core structure for the reaction and a set of functional groups. These functional groups are varied around the reaction core, where changing the functional groups will change the reaction’s activation barrier. Under this formulation, an algorithm can then search through the space of reactions to identify one with the desired activation barrier. There are many chemical problems in which an efficient search procedure would be highly desirable and could reduce waste: controlling reactivity or selectivity during a chemical synthesis,^33,34^ ensuring that a covalent drug has an optimal reactivity,^35,36^ or avoiding a potentially toxic side effect in a drug molecule if its reactivity were too high.^37^

Under our new formulation, we tested several algorithms to search spaces of reactions. Our most successful and thus recommended approach is a simple yet effective ML-based algorithm, which iteratively selects reactions that are predicted to have barriers closer to the target value. When using rough estimates of the reaction barriers, calculated at a lower level of theory compared with the target values, as an input feature, we found the performance of this algorithm to improve. The use of these approximate activation barriers appears to guide the ML model’s predictions in slightly better directions, allowing the algorithm to sample from more refined regions of the reaction space and thus speeding up its location of a target barrier. We also compare this approach with a simple baseline random search, two techniques based on local search, a genetic algorithm and Bayesian optimization, and find our technique to be the most successful.

Our proposed reformulation is also found to be effective over a range of activation barrier data sets, with a diverse set of reaction mechanisms. We present results from a new data set of aza-Michael additions (a reaction we select for its importance to toxicology^37,38^ and the creation of target covalent inhibitors^35,36,39,40^), E2 and S_N_2 reaction barriers,^22^ as well as a set of dihydrogen activations on derivatives of Vaska’s complex from Friederich et al.^20^ The latter data set was chosen as a validation experiment to ensure that our reformulated approach was capable of handling complex and highly relevant catalytic reactions.

Our experiments demonstrate that our approaches for identifying reactions with desired target barriers within chemical accuracy are very successful. In particular, when the target barrier lies within the densest regions of the distribution of barriers for a given data set, most approaches are consistently able to quickly identify a reaction with a barrier within 1 kcal mol^–1^ of the target values. However, our recommended ML approach was also found to be effective for finding the more challenging barrier targets at the tail ends of the barrier distribution, using merely double-digit numbers of data points during the search process. Compare this with previous studies of ML to predict activation barriers, which typically use hundreds,^16,18,20^ thousands,^19,41,42^ or tens of thousands^17,21^ of barriers calculated at a high level of theory.

## 2 Computational Methods

### 2.1 Barrier Data Sets

#### 2.1.1 Aza-Michael Addition

**Figure 1:**
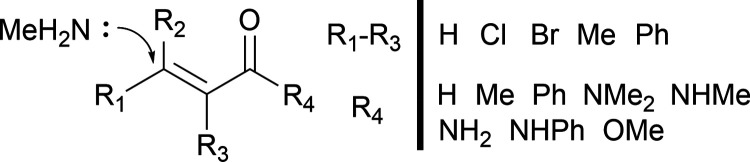
R-group placement scheme for the data set of Michael acceptors reacting with methylamine. The scheme on the right side shows all of the possible functional groups that may occupy each of the positions shown on the left side of the vertical black bar.

Figure 1 shows the scheme that was used to generate the structures of 1000 Michael acceptors and the 1000 corresponding transition states for their reactions with methylamine. The “R-Group Creator” and “Custom R-Group Enumeration” tools from Schrödinger’s Maestro v12.5^43^ were used for this structure generation, and following this, the reactants and constrained transition states were conformationally searched with the MMFF force field^44^ using the “Conformational Search” tool from Schrödinger’s MacroModel v12.9.^45,46^ Our choice of force field was due to previous findings that the MMFF force fields are among the most successful for predicting the conformations of organic molecules.^47,48^ The lowest-energy conformers of each of the reactants and transition states predicted by MMFF were retained for further quantum chemical calculations.

For this data set of aza-Michael addition reactions, we select the PM6 semiempirical method to calculate the approximate, low-level barriers that are used as an input feature to the ML model in our main search algorithm. Single-point energies using the PM6 method and geometry optimizations at the ωB97X-D/def2-TZVP level of theory were performed on all of the MMFF structures using Gaussian 16, Revision A.03,^49^ and Revision C.01.^50^ All DFT and semiempirical calculations were performed in the IEF-PCM (water)^51^ implicit solvent model due to its previous use in reaction modeling of systems similar to those of this data set.^3,37,38^ All 1000 transition states and reactants were successfully optimized with the chosen DFT method. Further information on the aza-Michael addition data set, including plots of the DFT barrier distribution and the correlation between the DFT and PM6 barriers, is found in the Supporting Information, Section S1.1.

#### 2.1.2 Dihydrogen Activation on Vaska’s Complex

**Figure 2:**
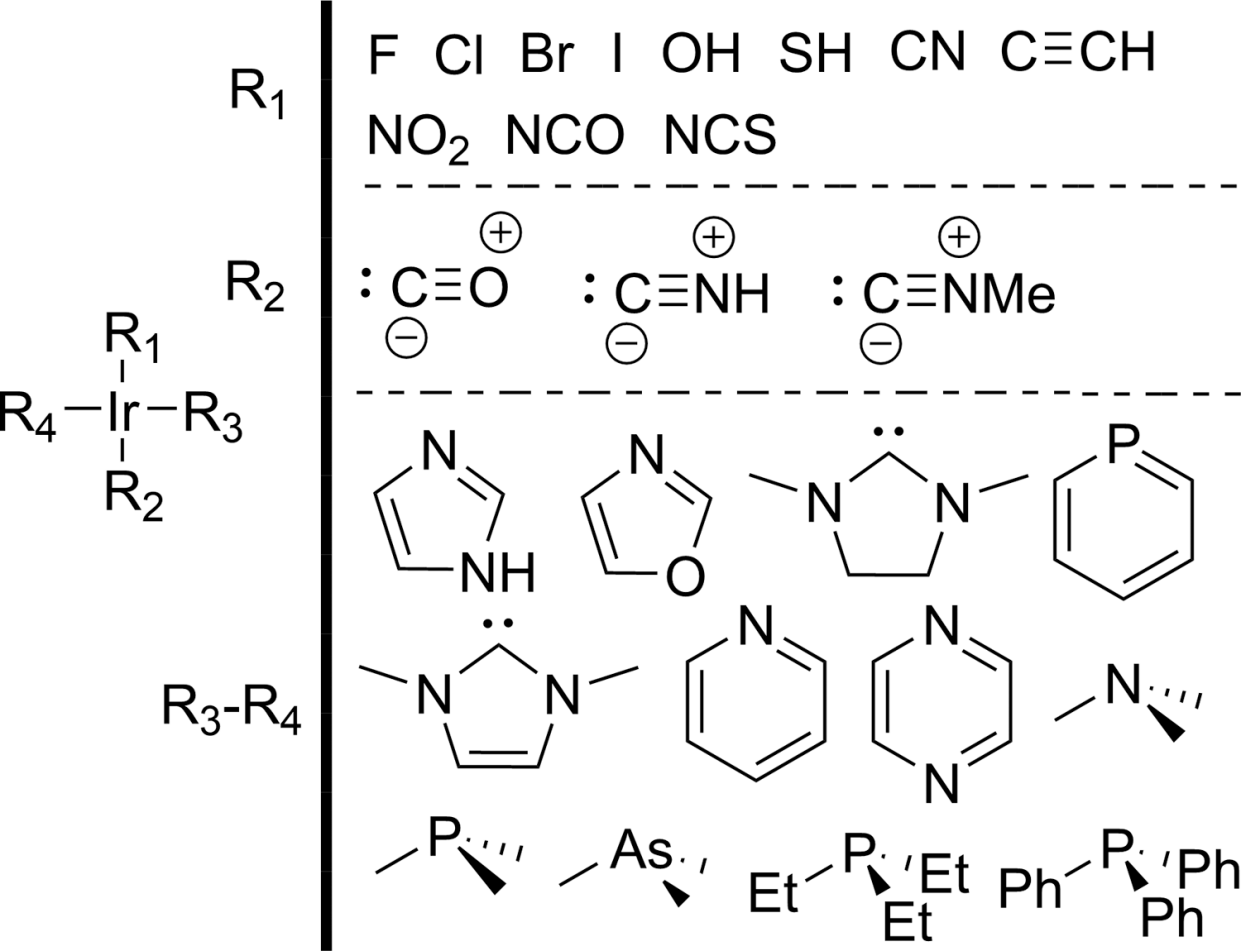
R-group placement scheme for the set of derivatives of Vaska’s complex by Friederich et al.^20^ The scheme on the right-hand side shows all of the possible functional groups that may occupy each of the positions shown on the left-hand side of the vertical black bar.

The data set of reaction barriers for dihydrogen activation on derivatives of Vaska’s complex by Friederich et al.,^20^ available from ref (52), was also utilized in this work. The enumeration scheme used by Friederich et al. for the R-groups attached to the iridium core of Vaska’s complex is shown in Figure 2. This scheme allows for the generation of a total of 2574 catalytic reactions once the symmetry of the catalyst is accounted for (R3 and R4 may be interchanged, and the reaction remains the same). 1947 dihydrogen activation barriers calculated at the PBE/def2-SVP level of theory including Grimme’s D3 dispersion correction^53^ were available from this data set, which gave a convergence rate of approximately 75.6%. Full details of the data set generation may be found in the original work by Friederich and co-workers.^20^

In this work, we also calculated single-point energies of the 1947 reactant and transition state structures that were available from this data set at the SVWN/def2-SVP level of theory (this LDA functional is at a lower rung and has cheaper computational cost compared with the PBE functional used to calculate the “accurate” barriers for this reaction) using Gaussian 16, Revision C.01.^50^ The approximate LDA activation barriers were calculated as the differences between the electronic energies of the reactants and transition states and are later used as the low-level barrier input feature to the ML model in our main search algorithm. For the 24.4% of the possible structures that were unavailable from this data set, their low-level LDA barriers were imputed using our selected Ridge regression model (see also Section S3 in the Supporting Information), trained on the set of LDA barriers calculated from the 75.6% of the possible reactions with coordinates available. Plots of the distribution of the accurate barriers and the correlation with the low-level LDA barriers may be found in the Supporting Information, Section S1.2.

#### 2.1.3 E2 and S_N_2

**Figure 3:**
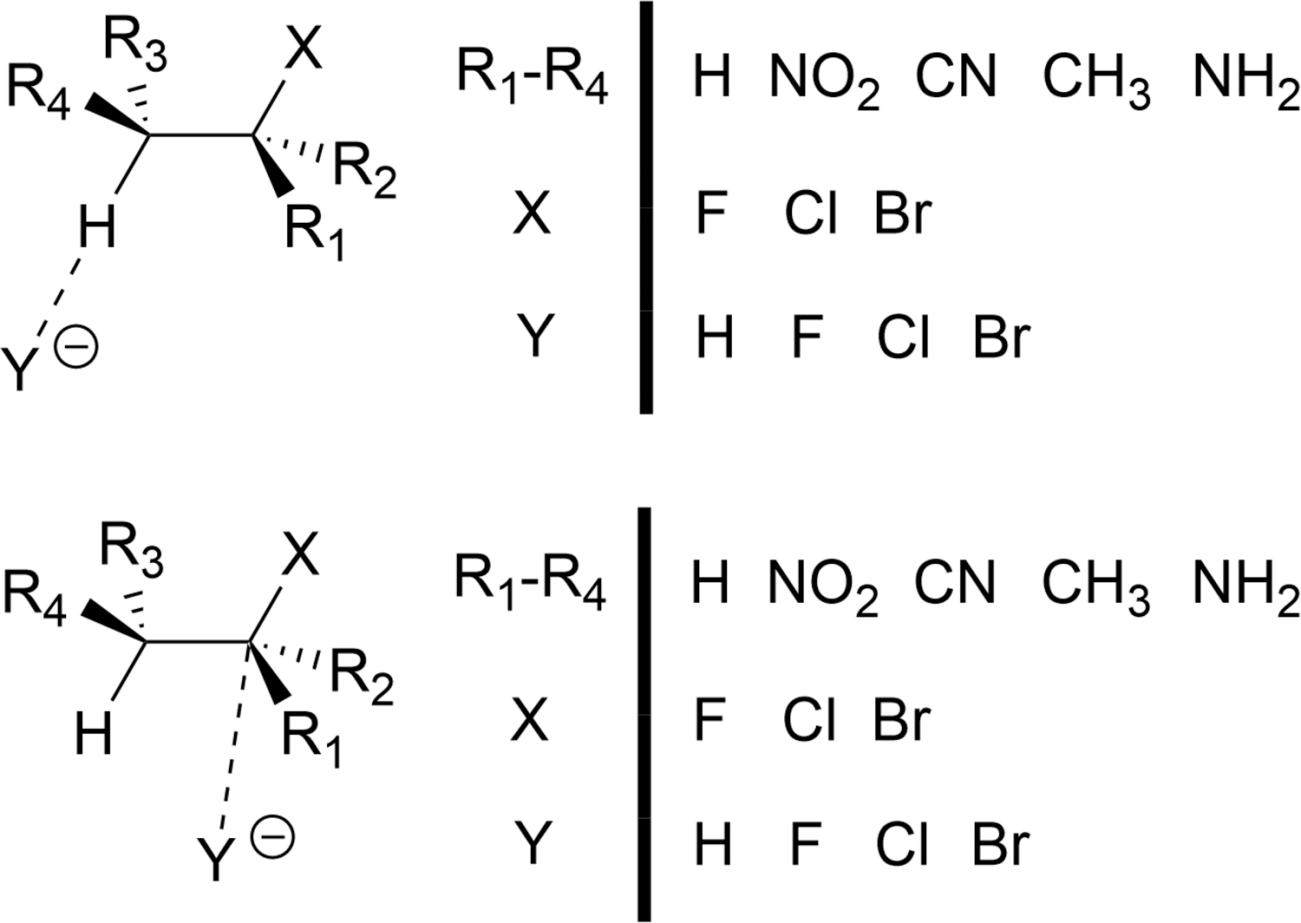
R-group, leaving group, and nucleophile placement scheme for the data sets of E2 (top) and S_N_2 (bottom) reactions by von Rudorff et al.^22^ The schemes on the right side show all of the possible functional groups that may occupy each of the positions shown on the left side of the vertical black bars.

The data sets of E2 and S_N_2 reaction activation barriers that were created by von Rudorff et al.^22,54^ were also generated via an enumeration scheme, and are therefore suitable for this work. The R-group, leaving group (X), and nucleophile (Y) placement schemes for the E2 and S_N_2 reactions are shown in Figure 3. These generation schemes allow for a total of 3900 distinct reactions to be generated after accounting for the symmetry of the systems (when R1 and R2 are interchanged simultaneously with R3 and R4, the reaction remains the same).

In this work, we treat the MP2/6-311G(d) E2 and S_N_2 barriers as our expensive, target level of theory and the HF/6-311G(d) barriers that are also available from this data set as the low-level barriers. Once symmetry duplicates are removed, this data set provides 1000 E2 and 1720 S_N_2 reaction barriers, all at both levels of theory, giving convergence rates of 25.6 and 44.1%, respectively. Full details of the E2 and S_N_2 data set generation may be found in the original work by von Rudorff and co-workers.^22^ As for the dihydrogen activation data set, we impute the missing lower-level barriers with our selected Ridge regression model, trained on the full set of HF/6-311G(d) barriers for each reaction. Visualizations of the MP2 barrier distributions and their correlation plots with the HF barriers are found in the Supporting Information, Section S1.3.

### 2.2 Machine Learning Search Algorithm

**Figure 4:**
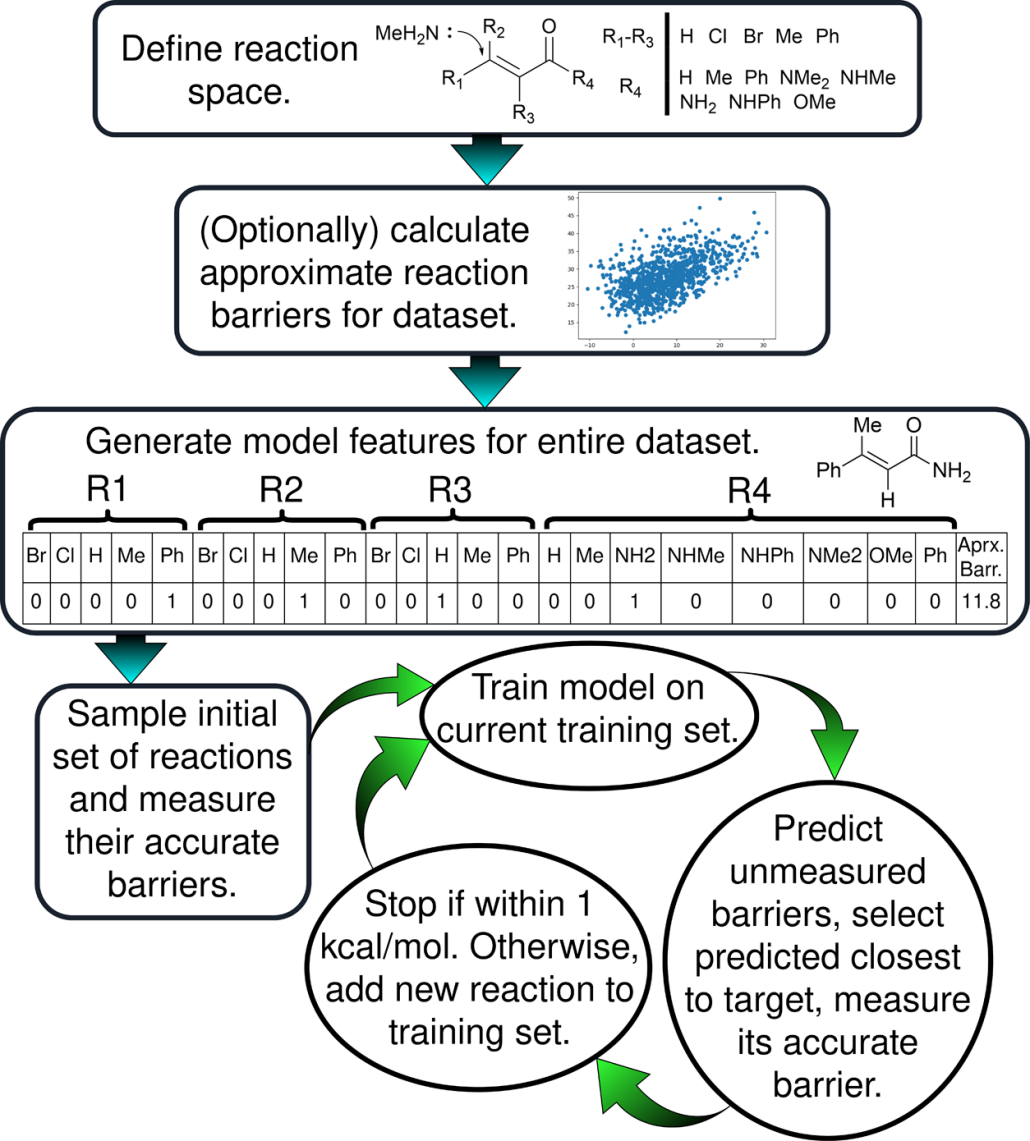
Overall scheme for the ML barrier search procedure in this work. The “generate model features for entire data set” box shows the representation vector used in this work for an example Michael addition reaction: a one-hot encoding of its R-groups concatenated with an approximate, low-level calculated barrier. Each column in this representation vector corresponds to one input feature for the ML model, and these features are generated for the whole data set of reactions before the search process can begin. Blue, straight arrows represent the initialization stages that are carried out before the iterative ML search (green, curved arrows).

Our ML search strategy is based on the iterative training of an ML model and using that model to select the most promising candidates from the pool of reactions without measured barriers. The procedure begins by randomly selecting five reactions from the defined search space, measuring their activation barriers, and training an ML model to predict the barriers of the remaining unmeasured set. Before the model is trained on the full set of measured reaction barriers, the hyperparameters for the model are selected by a small grid search according to those that minimize the mean absolute error from 5-fold cross-validation. The ML model is then made to predict the barriers of all of the unsampled reactions, and the reaction predicted to have a barrier closest to the target value has its activation barrier measured and is added to the “training” set. In the case that the predicted target reaction is unavailable due to calculation or experimental failure, that reaction is discarded from the pool of unsampled reactions, and the next most closely predicted reaction is measured and added to the training set. The model is then retrained with the augmented training set and the process repeats until a satisficing reaction with a barrier within 1 kcal mol^–1^ of the target value is obtained. Figure 4 also shows an illustration of this procedure in full.

The representation we use for the input to the ML models in this work is primarily composed of a one-hot encoding of its interchangeable functional groups. That is, each position in the reacting structures that may be substituted with a range of functional groups is assigned a bit vector, where each bit corresponds to one of the possible groups that may occupy that position (a value of zero indicates absence, one indicates presence). The bit vectors for each position are concatenated together to produce the representation of each reaction. In previous works predicting activation barriers,^16,18^ it was observed that approximate activation barriers calculated at a lower level of theory are extremely useful to the predictions of higher-level barriers. Therefore, in addition to the one-hot encoding, we tested as another input feature the approximate activation barriers of each of the reactions in the search space, as calculated with various lower levels of theory (see Section 2.1) or imputed with the same ML model used to search the reaction space where the structures are unavailable. The combination of one-hot encoding and low-level calculated barriers provides a powerful, yet compact reaction representation, which reduces the effects of the curse of dimensionality and therefore means that the ML model requires less data to train effectively and is less likely to overfit.

In order to make a principled selection of the particular ML model that would be used to search the reaction spaces in this work, we performed a small test of their predictive performances when trained on data sets of the tiny sizes they are expected to handle under our reformulation. Six ML models from the Python library scikit-learn^55^ (linear regression (LR), ridge regression (RR), random forest regression (RFR), gradient boosting regression (GBR), kernel ridge regression (KRR) and Gaussian process regression (GPR)) were selected and trained on 30 randomly selected data points from each of our data sets and the mean absolute errors of their predictions on the training set and the remaining “test” set were recorded. These tests were repeated five times, and we report the mean errors. The results of this test (in which we find ridge regression to be the overall best model) are reported in the Supporting Information, Section S3, along with the results of a similar experiment in which we use more chemically inspired input features instead of the one-hot encoding. However, we do not find these different features to generally improve the performance of our models in this context. The hyperparameters of the models were tuned before final training using a grid search, with selection based on the minimal mean absolute error from 5-fold cross-validation. The hyperparameter ranges used for each of the models are reported in the Supporting Information, Section S4.

To address the timings of each of the computational processes in this work, we emphasize that the time required for the entire search process (including model training and hyperparameter tuning in the supervised learning case) is completely eclipsed by the time for the high-level activation barrier calculation (in particular, the transition state optimization for our quantum mechanically calculated barriers). The time required for the optimization of a single transition state varies but is typically on the order of hours for the reacting species considered in this work. The searching code for a given reaction, on the other hand, takes a few minutes to complete, at most. Therefore, in practice, the major bottleneck when applying the methods presented in this work will be the determination of the small number of reaction barriers.

For each data set, we test our algorithms on a range of target values from across the barrier distributions. Starting from the minimum barrier reaction as a target value, we select the next target reaction as the one that is closest to having a barrier 2 kcal mol^–1^ higher than the previous target until the maximum barrier is reached. This provides a set of target values covering the entire range of the barrier distribution, each separated from the last by approximately 2 kcal mol^–1^. For each target barrier, we repeat the search experiments 25 times with a different initial random sample in each repeat. The metrics we report in this main paper are based on the mean numbers of sampled reactions over the 25 repeats.

In addition to this ML-based search algorithm, we test five other algorithms: a random search, two methods based on local search, Bayesian optimization, and a genetic algorithm (as well as a variant of our main algorithm that does not use the low-level barriers as an input feature), but these do not perform as well as our main ML algorithm discussed here. We report the workings of these additional five algorithms in the Supporting Information, Section S2.

## 3 Results and Discussion

### 3.1 Search Algorithm Performances

In the Supporting Information, Section S3, we report the results from our ML model assessment, in which we find that the ridge regression model showed the best balance between performance on held-out test data and lack of overfitting to the extremely small data set of 30 samples, and therefore we selected it as the ML model for the use in our main algorithm.

We assess our search algorithms by the number of reactions each required to sample before obtaining a reaction with a barrier within 1 kcal mol^–1^ of a target value. In the Supporting Information, Section S5, we show the means of these numbers averaged over the 25 repeats for all of the target barriers for each data set. In this section of this manuscript, we report these mean sample numbers averaged over different regions of the barrier distributions, to show how the performances of the different algorithms change when the target value moves to more challenging regions of the distributions.

Table 1 shows the mean numbers of reactions that are sampled by each of our algorithms, averaged over all of the target values for each data set. Our ML search procedure outperforms all of the other algorithms on each data set. It finds reactions within 1 kcal mol^–1^ of the target value with fewer samples than any of the other algorithms. This approach is followed closely in performance by Bayesian optimization, which requires very similar numbers of sampled reactions, although it seems that the additional leveraging of the predicted uncertainty does not offer a great advantage in this particular context.

**Table 1:** Mean Numbers of Sampled Reactions Required to Obtain Barriers within 1 kcal mol^–1^ of a Target Value, Averaged over all of the Target Values for Each Data Set

data set (% complete)	random search	local search	guided local	ML (no barr.)	ML search	bayes. opt.	genetic alg.
MA (100%)	167.11	37.96	26.03	16.78	**13.39**	13.50	63.16
H_2_ (75.6%)	318.94	132.85	259.24	39.09	**9.69**	10.30	189.22
S_N_2 (44.1%)	923.27	470.05	449.67	134.58	**47.75**	49.48	373.64
E2 (25.6%)	912.72	503.81	653.97	221.04	**100.24**	116.33	577.08

Rerunning the same ML search procedure with the exclusion of the low-level calculated barriers as input features (column titled “ML (no barr.)”) shows that the algorithm still performs well when the completion rate of the accurate barrier data set is high (i.e., in the aza-Michael addition and dihydrogen activation data sets). This suggests that one may implement a strategy in which the sampling procedure begins without using the low-level barriers as an input feature, and after a small number of samples, if the convergence rate of the accurate barriers is determined to be too low, the low-level barriers may then be calculated. This would allow for the avoidance of the calculations of the low-level barriers, should the convergence rate be high enough that the algorithm will perform similarly with or without that feature.

Also seen in Table 1 is that the local search and genetic algorithms perform notably worse than the model-based algorithms, and they are therefore not our recommended approaches for this problem reformulation. However, all of our tested algorithms outperform a totally random search, reassuring us that a principled approach to searching reaction spaces is more effective for our reformulation than the blind sampling of reactions.

We also observe that the smallest typical number of samples required to obtain a barrier close to the target value is found for the dihydrogen activation data set, which is most likely due to the very strong correlation between the set of PBE reaction barriers we treat as our target level of theory and the lower-level LDA barriers (see also Figure S2 in the Supporting Information). However, it is also seen that the S_N_2 and (in particular) the E2 reactions require significantly more samples to obtain the target barriers. This is most likely due to the very low convergence rates of the barriers in the two data sets (25.6% and 44.1% for E2 and S_N_2, respectively), meaning that there is a very high chance in each that any selected data point will not have a barrier available and thus the model will not be trained on this data and will be unable to leverage its information.

However, the sample numbers averaged over the entire set of barrier targets do not quite tell the whole story in terms of how the performances of the various algorithms vary as the target value moves to different regions of the barrier distributions. Table 2 shows the mean numbers of samples averaged over all of the target values except the highest five and lowest five (or highest three and lowest three for the dihydrogen activation data set since the range of its barrier distribution is much smaller than the other data sets). The algorithms now all perform more similarly, showing much more practically achievable numbers of sampled reactions. This better performance in the more central, denser regions of the barrier distribution is explained by the higher numbers of reactions with barriers close to these target values. For target barriers in regions of the barrier distribution with greater sample densities, there is a much higher chance that a randomly chosen reaction will have a barrier closer to the target value. This is most easily seen from the dramatic reduction in the averaged sample numbers for the random search seen in Table 2 compared with Table 1.

**Table 2:** Mean Numbers of Sampled Reactions Required to Obtain Barriers within 1 kcal mol^–1^ of a Target Value, Averaged over All except the 5 Highest and 5 Lowest Target Values (or 3 Highest and 3 Lowest in the Case of the Dihydrogen Activation Data Set, due to Its Shorter Barrier Range)

data set (% complete)	random search	local search	guided local	ML (no barr.)	ML search	bayes. opt.	genetic alg.
MA (100%)	18.95	14.71	14.75	9.57	9.46	**9.00**	17.75
H_2_ (75.6%)	21.08	24.45	23.79	11.14	**9.14**	9.75	13.50
S_*N*_2 (44.1%)	257.59	106.72	87.97	52.65	**33.41**	35.42	128.55
E2 (25.6%)	211.47	113.91	147.59	92.98	**70.37**	86.73	160.57

Therefore, the most important test of our algorithms is how many samples they require to find the reactions with the minimum and maximum barriers in each of the data sets. Table 3 shows the mean numbers of samples averaged over the five highest and five lowest target barriers for each data set (except for the dihydrogen activation data set, for which we average over the three highest and three lowest target barriers due to the smaller range of its barrier distribution). Again it is seen that our ML-based search approach using the low-level calculated barriers as an input feature requires the smallest numbers of sampled reactions, with Bayesian optimization using the same features following very closely behind. Our ML algorithm when not using the low-level barriers as a feature also performs quite closely for the aza-Michael addition data set (for which the completion rate is 100%). This again suggests that the low-level barriers do not necessarily have to be calculated when the convergence rate of the accurate barriers is high enough. We observe that the performances of these two algorithms do not deteriorate to as great an extent as our alternative search algorithms. For the aza-Michael addition and dihydrogen activation data sets, our ML algorithm is still able to find reactions with barriers within 1 kcal mol^–1^ of these most challenging target values within very reasonable numbers of samples. In the E2 and S_N_2 data sets, it is more difficult for our algorithm to locate reactions with target barriers close to the tail ends of the distributions, which is again very likely due to the low convergence rates of the barrier calculations in these data sets. However, our proposed ML method still shows the best performance out of all our algorithms for these challenging data sets.

**Table 3:** Mean Numbers of Sampled Reactions Required to Obtain Barriers within 1 kcal mol^–1^ of a Target Value, Averaged over the 5 Highest and 5 Lowest Target Values (or 3 Highest and 3 Lowest in the Case of the Dihydrogen Activation Data Set, due to Its Shorter Barrier Range)

data set (% complete)	random search	local search	guided local	ML (no barr.)	ML search	bayes. opt.	genetic alg.
MA (100%)	315.27	61.21	37.31	23.98	**17.32**	17.99	108.56
H_2_ (75.6%)	666.45	259.33	533.93	71.69	**10.34**	10.93	394.23
S_N_2 (44.1%)	3053.46	1632.69	1607.13	396.78	**93.64**	94.46	1157.91
E2 (25.6%)	2525.61	1400.59	1818.65	515.59	**168.92**	184.41	1535.06

At this point, some discussion of the generalities and limitations of our proposed approach is warranted. This reformulation has been tested on a diverse range of data sets, from toxicologically relevant aza-Michael addition to transition-metal-catalyzed dihydrogen activation, and is therefore suitable for a wide range of chemistries. Specifically, our approach is oriented toward the situation in which one is interested in the selection of a single reaction from a large set, which may be described by a core structure with variable surrounding R-groups (for example, Figures 1–3). The restriction to a search within a predefined set of reactions means that one no longer needs to be concerned whether the ML model is able to generalize beyond the specific domain of the data set at hand, thus avoiding this pitfall that is common to nearly all types of ML.

On the other hand, our most performant approach requires the calculation of low-level, approximate activation barriers for all of the possible reactions in the search space. However, the level of theory used may be quite low, only single-point energies on approximate transition state and reactant geometries are required (for example, for the aza-Michael addition data set, PM6 semiempirical calculations were performed on constrained MMFF geometries) and any missing low-level barriers may be imputed with an ML model once a great enough proportion of the data set has been covered (for example, as in the dihydrogen activation, E2 and S_N_2 data sets). Therefore, the calculations of these low-level barriers should not lead to an extremely problematic computational overhead and will be much cheaper than the determinations of the accurate reaction barriers. In addition, when the convergence rate of the accurate barriers for a given data set is high enough, the ML algorithm performs quite similarly when not using the low-level barrier as a feature (as for the aza-Michael addition and dihydrogen activation data sets). Therefore, the search algorithm could be implemented to switch to using the low-level barrier feature should the convergence rate of the accurate barriers be found to be too low after a small number of samples, and otherwise, the low-level barriers would not even need to be calculated.

The reformulation of the problem toward finding a particular reaction from a finite set of possibilities limits the eventual reaction that will be found to one that is within the initially specified set of reactions. This is in contrast to the idea of using ML models to predict the activation barriers of arbitrary chemical reactions with high accuracy and speed. However, training an ML model to make highly accurate predictions of barriers for a wide and useful variety of reactions requires an impractically large amount of expensive data. Therefore, we argue that approaches similar to those proposed here are worthy of further investigation. Specifically, a more effective use of ML and other computational techniques in the context of predicting activation barriers could create tools for efficiently identifying reactions with barriers that suit a particular purpose. Such methods could guide established experimental and computational methods rather than replacing them.

The decreases in the performance from our approach come when the completion rates of the data sets decrease. The E2 and S_N_2 data sets are only 25.6% and 44.1% complete, respectively, and give the lowest and second lowest performances of all of the data sets. This result is because the majority of the sampled data points during the ML search cannot be added to the training set, and therefore, the model is unable to leverage the information from that sample and cannot improve its predictions until a reaction with a new barrier value is obtained. However, we observe that the data set for which the best results were produced was for the dihydrogen activation reaction, which had a very strong correlation between its low-level barrier feature and the “accurate” barrier values. Therefore, in cases where such high failure rates are observed or expected, it may be prudent to obtain more expensive but more accurate lower-level barriers for the input feature, since they are more likely to be more strongly correlated with the accurate barriers.

Finally, in the Supporting Information, Section S6, we present additional analyses of the ML model used in the main search algorithm as the sampling iterations proceed. Analysis of feature importances shows that the low-level barrier features are most important and the model leverages the one-hot encoding features for small corrections to these approximate barriers. Visualizing the predictive performance of the model is as expected: the model’s performance on held-out test data improves as more data is sampled. Lastly, scrambling the input features severely deteriorates the performance of the search procedure at the challenging tail ends of the barrier distribution, indicating that the model was learning a meaningful relationship between the features and the activation barrier rather than simply overfitting and randomly guessing the correct reaction.

### 3.2 Case Study in Catalyst Optimization

Our results presented above give an empirical analysis of the efficiency and effectiveness of our method. However, an equally important consideration is its practical application. Therefore, as a final experiment, we wished to demonstrate how our reformulation may be put to use in a “lab-based” scenario. Suppose that one was performing a dihydrogen activation reaction, but the rate of reaction when using the current transition metal catalyst was either zero or much too slow and one wished to optimize the catalyst structure to reduce the reaction barrier and speed up the reaction as much as possible. To this end, we selected a “starting” reaction from the dihydrogen activation data set with a barrier in the upper region of the distribution at 20 kcal mol^–1^. We chose the target barrier of the reaction to be optimized to be the very minimum of the data set of dihydrogen activation reactions (1.6 kcal mol^–1^), and the process of identifying a reaction with this barrier was the same as our previous experiments.

The starting reaction was added to a data set of four randomly selected reactions (for which barrier measurements would first be performed either computationally or experimentally, in addition to the measurement of the starting reaction barrier). After adding an additional reaction to replace one from the initial sample that did not have a barrier available, this initial sample was used to train the ML model, which was then used to iteratively select the reactions that updated the training set. Table 4 shows the structures that were present in the initial sample and those that were selected until a reaction within 1 kcal mol^–1^ of the target value was obtained. Our algorithm sampled six additional reactions before finding a reaction with a catalyst that gave a barrier within 0.7 kcal mol^–1^ of the minimum barrier. It is a testament to the efficiency of our approach that this reaction turned out to have the second lowest barrier in the entire data set of dihydrogen activation reactions and that three of the iteratively selected reactions did not have accurate barriers available.

**Table 4:**
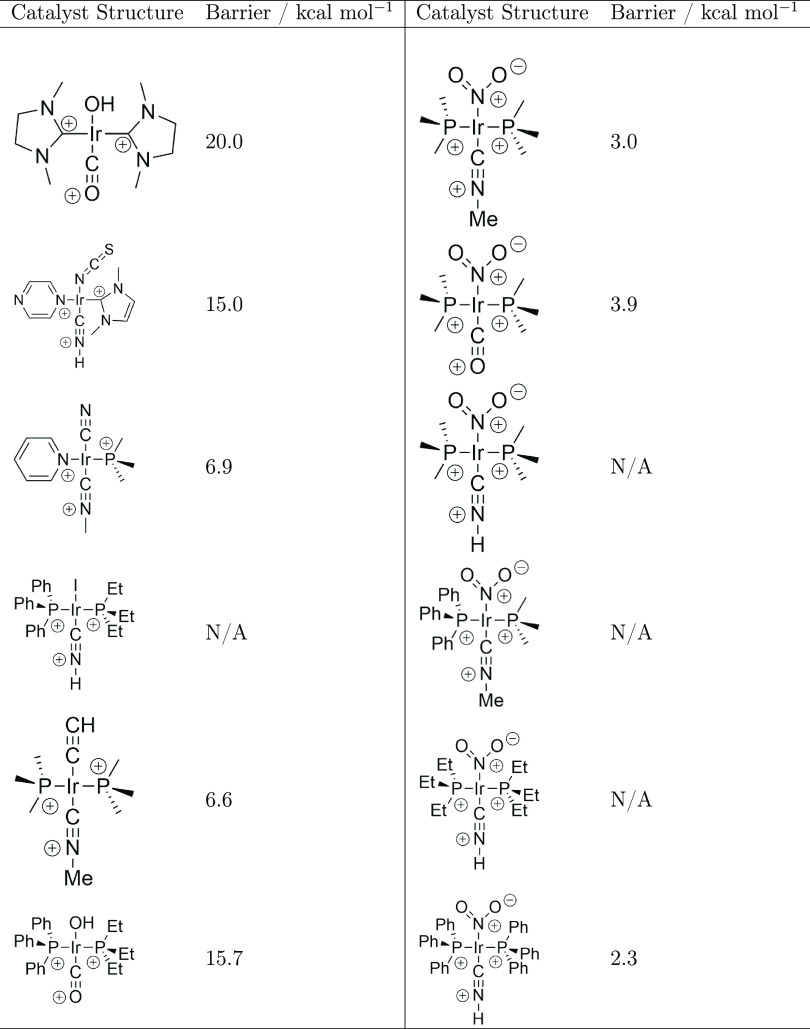
Left: The Catalyst Structures That Were Part of the Initial Sample of Reactions Used for the Initial Training of the Model in the ML Search Procedure Include the Starting 20 kcal mol^–1^ Catalyst and One of the Initially Selected Reactions That Did Not Have an Accurate Barrier Available. Right: The Catalyst Structures That Were Selected by the ML Search Algorithm, Where Descending the Column Gives the Order of Selection^a^

a N/A refers to reactions which were sampled but did not have accurate barriers available from the data set.

## 4 Conclusions

We have reformulated the problem of accurately predicting activation barriers for chemical reactions with ML toward a much more data-efficient process: finding a reaction with a desired target barrier from a prespecified set of possible reactions. In our formulation, each reaction differs from the next by modification of the functional groups around a reaction core. The overall aim of this reformulation is to significantly reduce the number of expensive, accurate barrier measurements that are required in order to obtain a reaction with desirable properties for any particular purpose.

We then proposed an ML-based approach for searching for these reaction spaces. In our data sets of aza-Michael addition and dihydrogen activation reactions, our algorithm was typically able to find reactions with barriers within 1 kcal mol^–1^ of a target value with less than 20 accurate barrier measurements. The performance of all our tested algorithms was somewhat reduced for the E2 and S_N_2 reactions due to the large number of missing accurate barriers from the original data sets. However, target barriers for the S_N_2 data set were still typically found within double-digit numbers of samples and even the E2 sampling requirements are much lower than previous uses of ML to predict activation barriers, the data sets for which often contain thousands of barriers.^17,19,21,41,42^ Additionally, in a case study in the use of our approach for the optimization of a transition metal catalyst for dihydrogen activation, within 12 barrier calculations, we were able to identify a reaction with a barrier within 1 kcal mol^–1^ of a target value, finding the second lowest barrier in the data set. This further demonstrated the efficiency of this reformulation in a particularly chemically complex scenario.

## Data Availability

Gaussian output files for the aza-Michael addition reactants and transition states and LDA single-point energies for the dihydrogen activation data set are available from the Unversity of Bath Research Data Archive (10.15125/BATH-01240).^56^ Code and other data are available from https://github.com/the-grayson-group/finding_barriers.

## Supporting Information Available

Additional details of data sets and plots of barrier distributions and correlations with low-level barriers; methods for all alternative search algorithms; results from ML model assessments with and without chemical features; hyperparamter ranges for all models throughout this work; tables of average numbers of samples used by each ML algorithm for each target barrier in each data set; additional results from analysis of machine learning model during search procedure: feature importances, mean absolute error scores, and results from using scambled features (PDF)
