# Correction for “Reformulating Reactivity Design for Data-Efficient Machine Learning”

**DOI:** 10.1021/acscatal.5c00556

**Published:** 2025-02-05

**Authors:** Toby Lewis-Atwell, Daniel Beechey, Özgür Şimşek, Matthew N. Grayson

A bug in the source code that was used for our original experiments in this work affected some of the results for the E2 and S_N_2 data sets in a minor way. These data sets taken from the literature contain reactions that are duplicated by symmetry, and therefore we removed these duplicated reactions from our training sets before running the barrier optimization experiments. However, a single missing line of code meant that the low-level barriers for these two data sets were not filtered along with the reactions and high-level barriers. This scrambled the relationship between the low- and high-level barriers, artificially reducing their correlation. Whereas the squares of the Pearson correlation coefficients between the low- and high-level barriers should have been 0.90 and 0.88 for the E2 and S_N_2 data sets, respectively, they were reduced to 0.56 and 0.65. With the fix implemented, we reran the experiments on the E2 and S_N_2 data sets and found that (as would be expected from the higher correlations) the data efficiency of the algorithms that use the low-level barriers is improved. In the original version of this work, values in the E2 and S_N_2 rows of the “ML Search” and “Bayes Opt.” columns of Tables 1, 2 and 3 should be changed to those given here.

**Table 1:** 

data set (% complete)	ML Search	Bayes. Opt.
S_N_2 (44.1%)	**28.15**	31.70
E2 (25.6%)	**78.12**	84.25

**Table 2:** 

data set (% complete)	ML Search	Bayes. Opt.
S_N_2 (44.1%)	**26.44**	31.17
E2 (25.6%)	**54.45**	64.28

**Table 3:** 

data set (% complete)	ML Search	Bayes. Opt.
S_N_2 (44.1%)	33.65	**33.42**
E2 (25.6%)	132.56	**130.17**

A very small change in the discussion is required to reflect these results. In the second paragraph on page 13511, the sentence that reads *“Again it is seen that our ML-based search approach using the low-level calculated barriers as an input feature requires the smallest numbers of sampled reactions, with Bayesian optimization using the same features following very closely behind”* should be changed to *“Again it is seen that our ML-based search approach using the low-level calculated barriers as an input feature requires the smallest numbers of sampled reactions, with practically no difference between the ML search and Bayesian optimization using the same features”*. Additionally, in this same paragraph, there are two sentences that are slightly unclear and should be updated. The sentence that begins with *“We observe that the performances of these two algorithms do not deteriorate ...”* should be changed to begin with *“We observe that the performances of the ML and Bayesian algorithms do not deteriorate ...”*. Finally, the last sentence of this paragraph that starts with *“However, our proposed ML method still shows the best performance ...”* should be changed to *“However, our proposed ML method of using low-level barriers still shows the best performance ...”*.

The results of all other algorithms and data sets are not affected, and none of the conclusions of the paper are affected. The proposed method of using low-level barriers remains equally valid, since even with artificially more weakly correlated low-level data, our approach is still more data-efficient compared with not using the low-level data.

In the Supporting Information, Section S1.3, on pages 4 and 5, we have corrected the Pearson correlation coefficients and mean absolute error metrics relating to the low- and high-level barriers of the E2 and S_N_2 data sets. We have also updated Figure S3 to show the correct relationships between the barriers of these data sets without the artificial decorrelation of the low-level barriers. In Sections S5.3 and S5.4, pages 19 and 22, we have updated Tables S11 and S13 to have the corrected values from the full results of the “ML Search” and “Bayes. Opt.” algorithms that used the low-level E2 and S_N_2 barriers. We further emphasize the advantage of our overall approach by including an additional section in the Supporting Information (new Section S7, pages 36–39) reporting the original results for the E2 and S_N_2 data sets.

## Supporting Information Available

Additional details of data sets and plots of barrier distributions and correlations with low-level barriers; methods for all alternative search algorithms; results from ML model assessments with and without chemical features; hyperparamter ranges for all models throughout this work; tables of average numbers of samples used by each ML algorithm for each target barrier in each data set; additional results from analysis of machine learning model during search procedure: feature importances, mean absolute error scores, and results from using scrambled features; original results for the E2 and S_N_2 data sets (PDF)

