# Supplementary material for: Correction for “Reformulating Reactivity Design for Data-Efficient Machine Learning”
Source: ACS Catal. 2025 Feb 5;15(4):2959–60. doi: 10.1021/acscatal.5c00556 (PMC11851427; doi:10.1021/acscatal.5c00556)
Supplement: Supplementary file 1 — cs5c00556_si_001.pdf [file cs5c00556_si_001.pdf]

Supporting Information for "Reformulating Reactivity Design for Data-Efficient Machine Learning"

Toby Lewis-Atwell,[a,b] Daniel Beechey,[b] Özgür Şimşek,[b] Matthew N. Grayson*[a]

E-mail:

a. Department of Chemistry, University of Bath, Claverton Down, Bath, BA2 7AY, UK

b. Department of Computer Science, University of Bath, Claverton Down, Bath, BA2 7AY, UK

*Corresponding author: M.N.Grayson@bath.ac.uk

Contents

S1 Further Dataset Details **3**

S1.1 Aza-Michael Addition 3

S1.2 Dihydrogen Activation on Vaska's Complex 4

S1.3 E2 and S_N2 4

S2 Details of Additional Algorithms **6**

S2.1 Random Search 6

S2.2 Local Search 6

S2.3 Guided Search 7

S2.4 Bayesian Optimization 8

S2.5 Genetic Algorithm 9

S3 Machine Learning Model Assessments **10**

S4 Model Hyperparameters **12**

S5 Sampling Numbers **13**

S5.1 Aza-Michael Addition 13

S5.2 Dihydrogen Activation 15

S5.3 S_N2 17

S5.4 E2 20

S6 ML Model Analysis **23**

S6.1 Feature Importances 23

S6.2 Model Performance 32

S6.3 Scrambled Features 34

S7 Old E2 and S_N2 Results with Scrambled Low-Level Barriers **36**

References **40**

S1 Further Dataset Details

S1.1 Aza-Michael Addition

Energetic data were extracted from the aza-Michael addition output files using the GoodVibes python library[1] with concentration set to 1.0 mol/L and temperature set to 298.15 K. The DFT activation barriers were calculated as the differences between the quasi-harmonic Gibbs free energies of the reactants and transition states from the GoodVibes output, for which Grimme's quasi-harmonic treatment of entropy[2] was used. The approximate semi-empirical activation barriers were calculated as the differences between the PM6 electronic energies of the MMFF reactant and constrained transition state geometries.

Figure S1a) shows the distribution of ωB97X-D/def2-TZVP barriers for the aza-Michael addition dataset. The barriers in this dataset range from 12.2 to 49.8 kcal mol^{-1}, with a mean value of 27.8 kcal mol^{-1} and a standard deviation of 5.36 kcal mol^{-1}. Figure S1b) shows a plot of the approximate PM6 activation barriers against the DFT barriers. The R^2 value between the DFT and PM6 barriers was 0.29 and the mean of the absolute errors was 20.0 kcal mol^{-1}.

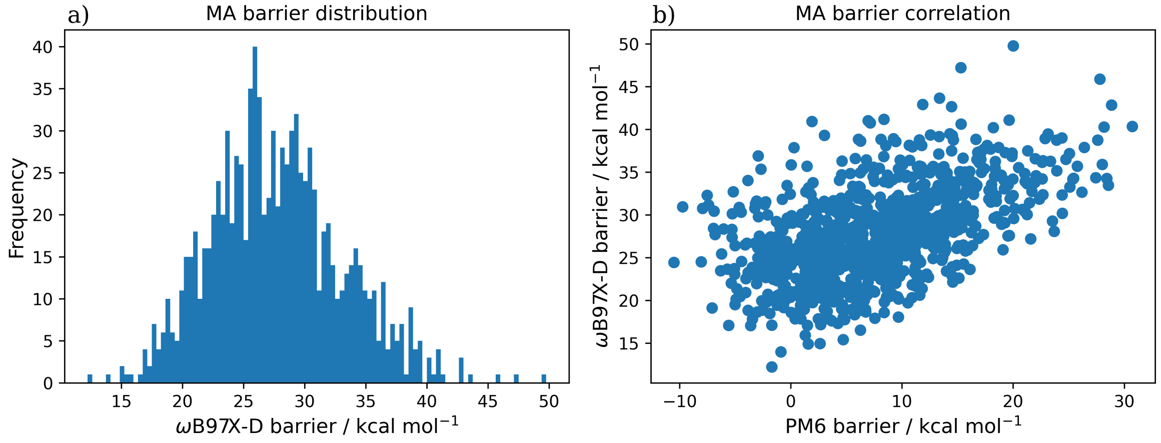

Figure S1: a) The distribution of the activation barriers of the aza-Michael addition (MA) dataset as calculated at the ωB97X-D/def2-TZVP level of theory. b) The correlation between the aza-Michael addition barriers calculated with single-point energies from the PM6 semi-empirical method and at the ωB97X-D/def2-TZVP level of theory.

S1.2 Dihydrogen Activation on Vaska's Complex

Figure S2a) shows the distribution of the barriers of this dataset; it has a minimum barrier at 1.6 kcal mol^{-1}, a maximum barrier of 25.6 kcal mol^{-1}, a mean value at 12.0 kcal mol^{-1} and a standard devition of 4.3 kcal mol^{-1}. Figure S2b) shows the correlation between the approximate LDA/def2-SVP barriers and the PBE/def2-SVP barriers. The R^2 value between the PBE and LDA barriers was found to be 0.93 and the mean of the absolute errors was 9.47 kcal mol^{-1}.

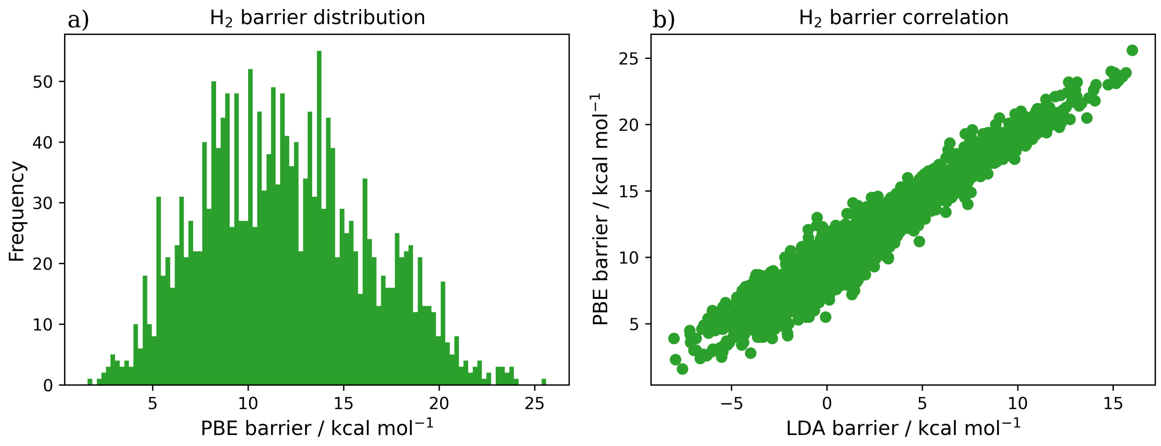

Figure S2: a) The distribution of the activation barriers of the dihydrogen activation (H_2) reaction on Vaska's complex as calculated at the PBE-D3/def2-SVP level of theory. b) The correlation between the dihydrogen activation barriers calculated at the PBE/def2-SVP and LDA/def2-SVP levels of theory.

S1.3 E2 and S$_N$2

Figure S3a) shows the distribution of MP2/6-311G(d) barriers for the E2 dataset. The barriers in this dataset range from −20.7 to 60.6 kcal mol^{-1}, with a mean value of 6.34 kcal mol^{-1} and a standard deviation of 12.2 kcal mol^{-1}. Figure S3b) shows correlation between the HF/6-311G(d) barriers and the MP2 barriers. The R^2 value between the MP2 and HF E2 barriers was found to be 0.90 and the mean of the absolute errors was 8.97 kcal mol^{-1}. Figure S3c) shows the distribution of MP2/6-311G(d) barriers for the S$_N$2 dataset. The

barriers in this dataset range from -42.9 to 62.4 kcal mol^{-1}, with a mean value of 21.7 kcal mol^{-1} and a standard deviation of 13.7 kcal mol^{-1}. Figure S3d) shows correlation between the HF/6-311G(d) barriers and the MP2/6-311G(d) barriers. The R^2 value between the MP2 and HF S_N2 barriers was found to be 0.88 and the mean of the absolute errors was 5.93 kcal mol^{-1}.

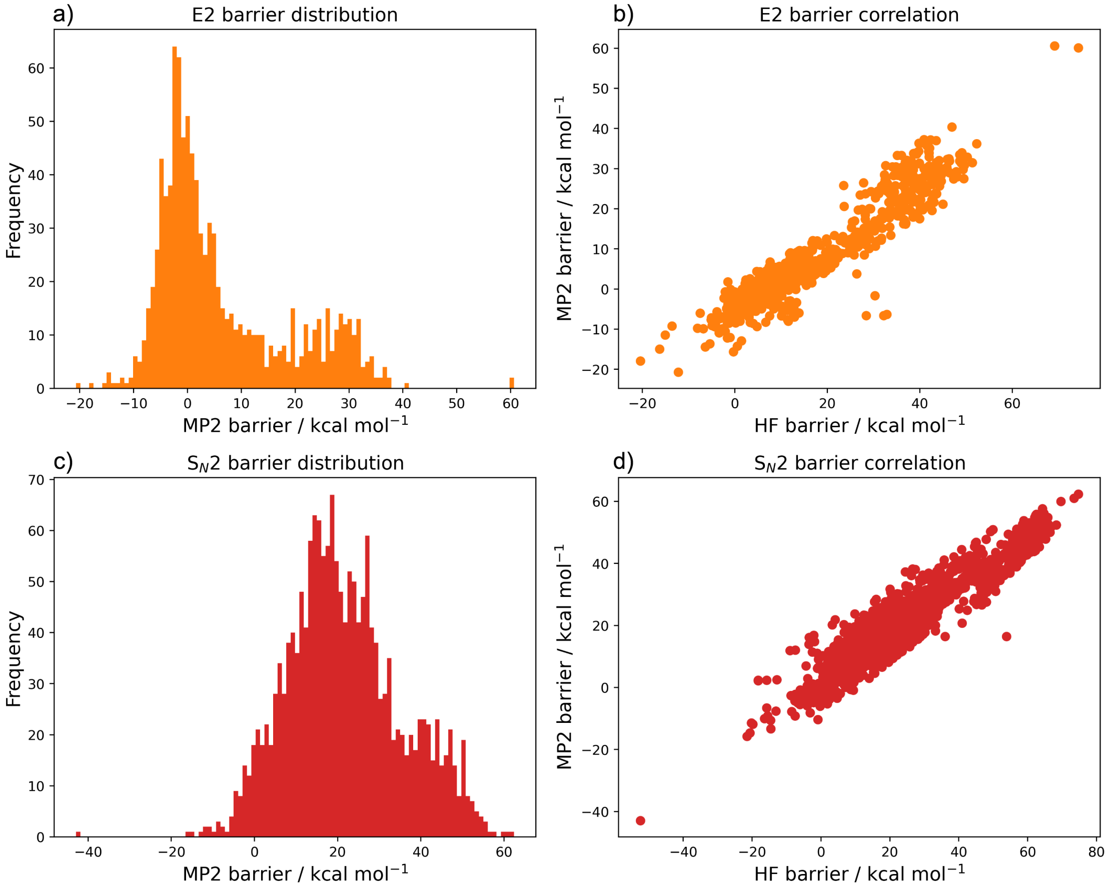

Figure S3: a) The distribution of the activation barriers of the E2 reaction as calculated at the MP2/6-311G(d) level of theory. b) The correlation between the E2 barriers calculated at the MP2/6-311G(d) and HF/6-311G(d) levels of theory. c) The distribution of the activation barriers of the S_N2 reaction as calculated at the MP2/6-311G(d) level of theory. d) The correlation between the S_N2 barriers calculated at the MP2/6-311G(d) and HF/6-311G(d) levels of theory.

We note that in these datasets are an appreciable number of unphysical negative MP2 barriers. However, they are still representative of a possible distribution of reaction activation barriers, but shifted downwards by approximately -20 kcal mol^{-1}, which makes no difference

to our algorithms.

S2 Details of Additional Algorithms

S2.1 Random Search

The experimental procedure for the random search starts with no barriers calculated. At each step, a new reaction is randomly sampled. When a sampled reaction's barrier is within 1 kcal mol^{-1} of the target barrier the procedure terminates and returns the number of reactions sampled. If a sampled reaction is not present in the dataset then its barrier is unknown and a new reaction is sampled, but the missing reaction still contributes to the number sampled. This procedure is repeated for several target barriers.

For each target barrier the experiments are repeated 25 times, with each repeat having a different order of reactions randomly sampled. The results report the mean and standard deviation over the 25 repeats. The random search acts as a baseline in our experiments.

S2.2 Local Search

A local search technique[3] is a more sophisticated alternative to a random search. To start, a random "root" reaction is sampled. Then, the set of "neighbour" reactions to the root is generated by changing the R group at each functional position to another R group. Figure S4 shows a subset of neighbours for a selected root. The neighbouring reaction whose barrier is closest to the target is then selected as the new root reaction and the procedure repeats. If no neighbouring reaction has a barrier closer to the target then a new root reaction is randomly sampled. The procedure terminates when a reaction is found whose barrier is within 1 kcal mol^{-1} to the target barrier, with the number of reactions sampled then returned.

If a reaction is not present in the dataset then it is ignored by the search but still included in the number of reactions searched. The local search procedure was repeated for several target barriers. For each target barrier experiments were repeated 25 times, with each repeat

having different initial root reactions sampled. The results report the mean and standard deviation over the 25 repeats.

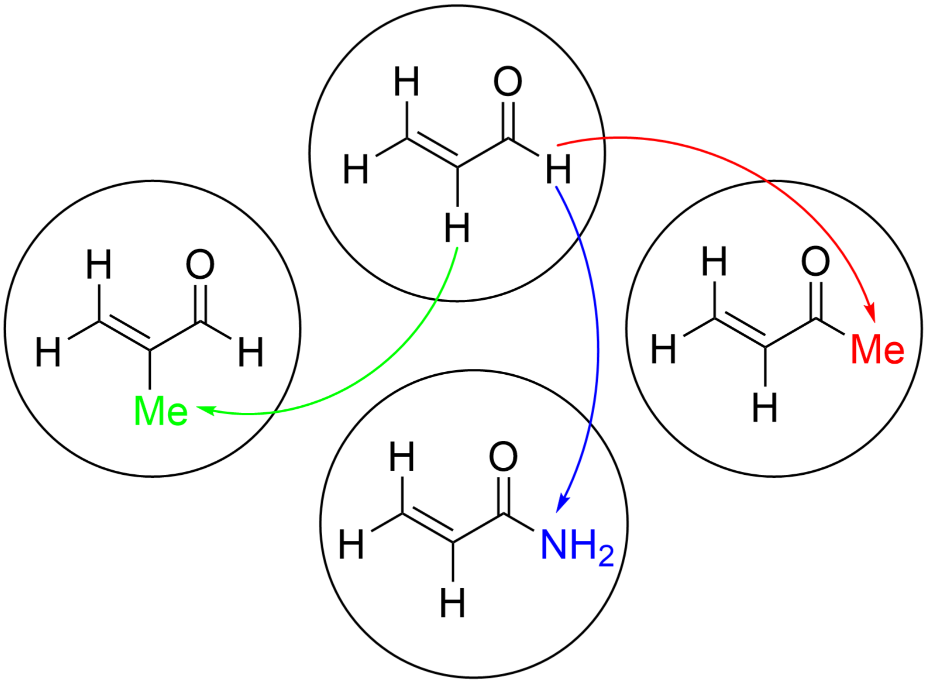

Figure S4: Three possible "neighbours" for a "root" reaction using the aza-Michael addition dataset. In each reaction node, the nucleophile (methylamine) is not shown, since it is the same for all reactions in the dataset. The sub-graph demonstrates how neighbours are generated by changing a single functional group of one reaction to create another.

S2.3 Guided Search

A search algorithm named Guided Search is also proposed, outlined here using terminology from the local search procedure introduced above. To start, a random root reaction is sampled. Then, the search selects the root's neighbouring reactions that were created by changing the R group at a single functional position. From this set, the reaction whose barrier is closest to the target (including the root) is selected as the new root. This procedure is repeated for the remaining function positions, at which point a new base is randomly sampled. The procedure terminates when a searched molecule's barrier is within 1 kcal mol^{-1} to the target barrier, with the number of molecules sampled returned.

If a reaction does not have a barrier available in the dataset then it is ignored by the search but still included in the number of reactions searched. The guided search procedure was repeated for several target barriers. For each target barrier experiments were repeated 25 times, with each repeat having different initial root reactions sampled. The results report the mean and standard deviation over the 25 repeats.

S2.4 Bayesian Optimization

The technique of Bayesian optimization is very similar to that of our ML search approach. Again, an initial sample of 5 reactions is used to train the probabilistic surrogate model (for which we used a Bayesian ridge regression model due to our selection of the ridge regression model for the ML search algorithm, see Section S3 and Section 2.2 of the main manuscript), and the same one-hot encoding and low-level barrier features were used as input to the model. Rather than selecting the reactions that were predicted to have barriers closest to the target values, our Bayesian optimization chooses the unsampled reaction with the maximum expected improvement (EI) value, shown in Eq. 1, as the next most promising reaction.[4,5]

$$\mathrm{EI}(x) = (\mu(x) - f(x*))\,\Phi\left(\frac{\mu(x) - f(x^*)}{\sigma(x)}\right) + \sigma(x)\phi\left(\frac{\mu(x) - f(x^*)}{\sigma(x)}\right) \tag{1}$$

Where $\mu(x)$ is the negative absolute difference between the predicted mean (i.e. the predicted barrier) from the probabilistic surrogate model and the target value for a reaction represented by the feature vector x, $\sigma(x)$ is the standard deviation from the model, $f(x^*)$ is the maximum of the negative absolute differences between the measured reaction barriers and the target value (it represents the "best" barrier sampled thus far), and Φ and ϕ represent the cumulative distribution function and the probability density function of the normal distribution respectively. The use of the expected improvement value in Bayesian optimization leverages the predicted uncertainty from the model, and thus introduces a greater degree

of exploration to the search compared with our largely exploitative pure-ML approach. In all other aspects, the Bayesian optimization proceeds in the same manner as our ML search algorithm.

S2.5 Genetic Algorithm

In our genetic algorithm, each reaction is represented by a bit vector, where a set of slices of that bit vector correspond to numbers in binary that represent the functional groups at each position in the reacting structures. To convert between the genetic algorithm's bit vectors and a vector of integers between 0 and the number of possible groups at each position (where each value corresponds to different group), the number's base is converted from binary to decimal, and it is scaled down to match the total number of allowed groups at a given position (since the number of bits required to represent one number may allow for values larger than the number of allowed groups at one position). The scaled (floating-point) numbers are then converted to integers using the procedure by Deep *et. al.* for mixed-integer genetic algorithms.[6] Thus, each bit vector proposed by the genetic algorithm may be converted to a list of numbers that corresponds to a specific reaction from our dataset's defined reaction space.

In order to keep the expense of running the genetic algorithm low (in terms of number of barrier measurements required to simply start the procedure), we set the population size to 20. After the initial random 20 bit vectors are decoded and have their corresponding barriers measured, a sample of the most promising reactions (as determined from the absolute difference between the reactions' barriers and the target value) undergo crossover (where bits to the right of a random crossover point in one bit vector are swapped with those from another) with a probability of 90% and mutation (random bits are swapped from zero to one or vice versa) with a probability of 5%. The procedure of crossover and mutation is repeated until a new generation of bit vectors has been created, and the algorithm terminates once a satisficing reaction with a barrier within 1 kcal mol^{-1} of the target value has been found.

For each target value, the genetic algorithm search was repeated 25 times, and the metrics reported here are based on the mean numbers of sampled reactions over the 25 repeats.

S3 Machine Learning Model Assessments

Tables S1 and S2 show the training and test mean absolute error scores (averaged over 5 random training sets), respectively, resulting from our assessment of several ML models to decide which was to be used for our search procedure. Table S2 shows that the models with the best performance on held out test data were RR and GPR, and they show very similar test performances across the datasets. However, the perfect scores on the training data from the GPR model in Table S1 show that this model had completely overfit to the tiny dataset of 30 samples. Therefore, we opted for RR as the ML model to be used in the search procedure due to its equally strong and more balanced performance when trained on these extremely small datasets.

Table S1: Mean values and standard deviations of mean absolute error scores (in kcal mol^{-1}) on the training set from 5 randomly sampled training sets of 30 data points.

Dataset	LR	RR	RFR	GBR	KRR	GPR
MA	0.94 ± 0.16	1.20 ± 0.11	1.17 ± 0.09	0.56 ± 0.96	1.10 ± 0.62	**0.00 ± 0.00**
H_2	0.03 ± 0.06	0.12 ± 0.12	0.48 ± 0.09	0.01 ± 0.01	0.03 ± 0.06	**0.00 ± 0.00**
E2	0.86 ± 0.31	1.04 ± 0.25	1.08 ± 0.10	0.32 ± 0.55	0.58 ± 0.53	**0.00 ± 0.00**
S_N2	0.74 ± 0.28	0.97 ± 0.46	1.76 ± 0.36	0.13 ± 0.14	0.35 ± 0.42	**0.00 ± 0.00**

Table S2: Mean values and standard deviations of mean absolute error scores (in kcal mol^{-1}) on the testing sets (all reactions not used in 30 data point training set) from 5 randomly sampled training sets.

Dataset	LR	RR	RFR	GBR	KRR	GPR
MA	3.29 ± 0.33	**2.60 ± 0.28**	3.36 ± 0.24	3.40 ± 0.28	3.22 ± 0.92	2.71 ± 0.31
H_2	2.41 ± 1.75	0.85 ± 0.11	1.19 ± 0.05	1.15 ± 0.12	0.87 ± 0.11	**0.80 ± 0.10**
E2	3.30 ± 0.35	**2.64 ± 0.15**	3.02 ± 0.07	3.20 ± 0.11	**2.64 ± 0.14**	2.65 ± 0.20
S_N2	4.15 ± 0.93	3.18 ± 0.33	4.08 ± 0.21	4.15 ± 0.14	3.01 ± 0.31	**2.90 ± 0.26**

We also test whether the use of alternative descriptors, calculated from the chemical structures of the reactants may help to improve the model's performance. To this end,

we repeated the experiment in which we train a set of ML models on 30 data points from each reaction dataset and measure their performances, but we replace the one-hot encoding features with descriptors calculated from RDKit's `CalcMolDescriptors` function.[7] This function calculated a wide range of chemical descriptors for the reactants and catalysts in the aza-Michael addition and dihydrogen activation datasets. These descriptors included the Gasteiger partial charges[8] for the core reacting atoms, descriptors of molecular connectivity,[9] Crippen calculated properties,[10] counts of various substructures and groups, and descriptors of molecular surface areas. The full list of descriptors and their references are available from[11]. We remove all descriptors that have the same value for all of the reactions in a dataset as well as those that produce NaN values. In total, this gave 127 features for the aza-Michael addition dataset and 111 features for the dihydrogen activation dataset. All non-count-based descriptors were scaled to zero mean and unit standard deviation. The low-level barriers were also included as a feature.

Table S3 and Table S4 show the train and test scores from this experiment respectively. As may be seen, these features do not lead to an improvement in the models' performances on the held-out test data compared with when using the one-hot encoding. This is most likely to be the case because the two descriptor sets are essentially representing the same information, but the one-hot encoding achieves this in a more dimensionally compact form compared with the RDKit descriptors. This shorter representation length reduces the effects of the curse of dimensionality and hence decreases the amount of overfitting in the one-hot models and thus improves their performance on unseen data.

Table S3: Mean values and standard deviations of mean absolute error scores (in kcal mol^{-1}) on the training set from 5 randomly sampled training sets of 30 data points using the alternate feature sets.

Dataset	LR	RR	RFR	GBR	KRR	GPR
MA	0.02 ± 0.05	2.28 ± 0.52	1.27 ± 0.16	1.46 ± 1.53	2.08 ± 0.33	**0.00 ± 0.00**
H_2	**0.00 ± 0.00**	0.14 ± 0.08	0.56 ± 0.07	0.01 ± 0.01	0.12 ± 0.07	**0.00 ± 0.00**

Table S4: Mean values and standard deviations of mean absolute error scores (in kcal mol^{-1}) on the held-out test set from 5 randomly sampled training sets of 30 data points using the alternate feature sets.

Dataset	LR	RR	RFR	GBR	KRR	GPR
MA	5.86 ± 1.92	3.45 ± 0.14	**3.22 ± 0.21**	3.69 ± 0.51	3.41 ± 0.20	4.02 ± 0.21
H_2	1.54 ± 0.44	**1.24 ± 0.16**	1.32 ± 0.10	1.35 ± 0.12	1.28 ± 0.14	3.31 ± 0.40

S4 Model Hyperparameters

Table S5 shows each of the hyperparameters and the corresponding values that were tuned during each of the grid searches, for all of the ML models used in this work, including the Bayesian ridge regression model used in the Bayesian optimization algorithm.

Table S5: *scikit-learn* ML model names and hyperparameters and values that were tested during grid searches for hyperparameter tuning.

Model	Hyperparameters
LinearRegression	"fit_intercept": [True, False]
Ridge	"alpha": [10**i for i in range(-7, 3)]
RandomForestRegressor	"n_estimators": [50, 100, 200]<br>"max_depth": [i for i in range(3, 36, 8)]
GradientBoostingRegressor	"n_estimators": [50, 100, 200]<br>"max_depth": [i for i in range(3, 36, 8)]<br>"learning_rate": [0.9, 0.1, 0.01, 0.001]
KernelRidge	"kernel": ["linear", "poly", "rbf", "sigmoid"]<br>"alpha": [10**i for i in range(-7, 3)]<br>"gamma": [10**i for i in range(-7, 3)]<br>"degree": [2, 3, 4]
GaussianProcessRegressor	"kernel": [RBF(), RationalQuadratic(), ExpSineSquared(), Matern(), RBF() + Matern(), RBF() + ExpSineSquared()]
BayesianRidge	"alpha_init": [10**i for i in range(-7, 3)]<br>"lambda_init": [10**i for i in range(-7, 3)]

S5 Sampling Numbers

S5.1 Aza-Michael Addition

Table S6: Mean values of the numbers of samples for each of the target values for the aza-Michael addition dataset with the random and two local search algorithms, averaged over 25 repeats. Metrics averaged over these values are reported in the main manuscript.

Barrier	Random Search	Local Search	Guided Local
12.23	472.56 ± 296.45	48.56 ± 14.02	23.52 ± 13.67
13.97	240.68 ± 183.57	47.6 ± 26.83	27.04 ± 4.98
16.52	88.16 ± 77.26	34.68 ± 12.39	18.04 ± 8.64
18.33	26.08 ± 19.5	24.0 ± 8.52	14.72 ± 3.62
20.24	20.04 ± 19.34	21.8 ± 17.62	12.4 ± 4.74
22.2	9.96 ± 10.31	15.16 ± 9.58	12.52 ± 6.94
24.23	8.32 ± 6.37	9.8 ± 7.99	8.6 ± 5.31
26.23	6.76 ± 5.12	7.68 ± 5.63	8.04 ± 3.94
28.22	5.0 ± 3.95	5.8 ± 5.15	8.52 ± 6.88
30.21	8.44 ± 8.64	9.04 ± 9.14	7.84 ± 3.87
32.21	10.44 ± 6.29	14.2 ± 9.34	12.88 ± 7.78
34.21	20.08 ± 16.74	16.0 ± 13.76	9.32 ± 7.23
36.24	22.36 ± 25.12	18.2 ± 13.66	13.92 ± 10.14
38.14	33.56 ± 33.31	20.76 ± 14.3	16.76 ± 7.66
40.2	64.56 ± 47.16	30.48 ± 18.05	49.08 ± 31.99
42.66	312.88 ± 209.41	55.44 ± 29.46	29.8 ± 16.65
43.64	312.88 ± 209.41	54.2 ± 25.69	29.64 ± 16.74
45.86	533.56 ± 345.53	149.72 ± 93.79	42.96 ± 33.02
47.22	529.64 ± 255.98	99.64 ± 69.3	88.72 ± 51.56
49.76	616.2 ± 272.48	76.48 ± 49.24	86.24 ± 50.94

Table S7: Mean values of the numbers of samples for each of the target values for the aza-Michael addition dataset with the ML, Bayesian and genetic algorithms, averaged over 25 repeats. Metrics averaged over these values are reported in the main manuscript.

Barrier	ML (no barr.)	ML Search	Bayes. Opt.	Genetic Alg.
12.23	25.72 ± 13.08	17.60 ± 7.92	15.20 ± 4.49	108.40 ± 56.45
13.97	27.80 ± 16.72	20.24 ± 11.84	18.52 ± 8.64	90.36 ± 42.25
16.52	16.64 ± 8.47	13.52 ± 8.04	14.44 ± 8.47	50.44 ± 27.38
18.33	10.92 ± 5.12	10.72 ± 4.77	11.52 ± 4.20	26.48 ± 18.53
20.24	7.92 ± 2.91	9.16 ± 4.43	7.56 ± 2.93	14.84 ± 12.17
22.2	7.72 ± 2.41	9.68 ± 4.34	9.48 ± 4.63	11.08 ± 7.40
24.23	9.56 ± 4.19	7.88 ± 2.45	8.64 ± 4.06	6.88 ± 4.78
26.23	7.04 ± 1.48	8.68 ± 4.29	9.44 ± 5.39	5.76 ± 5.52
28.22	7.68 ± 2.82	7.64 ± 2.84	6.96 ± 1.66	8.88 ± 8.34
30.21	6.80 ± 2.04	7.96 ± 4.28	7.24 ± 2.70	6.88 ± 6.53
32.21	9.32 ± 5.01	11.04 ± 7.39	9.88 ± 6.25	8.92 ± 8.84
34.21	8.80 ± 3.81	9.44 ± 4.20	8.92 ± 4.00	11.60 ± 8.85
36.24	11.44 ± 8.66	11.52 ± 6.36	10.00 ± 5.58	27.80 ± 24.97
38.14	11.76 ± 5.52	9.88 ± 3.52	10.64 ± 3.75	26.52 ± 18.02
40.2	15.60 ± 6.47	10.92 ± 7.48	8.84 ± 3.40	63.16 ± 39.99
42.66	25.12 ± 13.33	11.56 ± 8.80	14.24 ± 7.25	97.32 ± 55.96
43.64	23.32 ± 11.40	11.12 ± 3.89	14.44 ± 6.69	97.40 ± 56.09
45.86	27.00 ± 17.05	18.68 ± 14.90	24.00 ± 15.93	197.08 ± 95.94
47.22	43.20 ± 18.11	35.92 ± 16.82	35.08 ± 12.96	209.32 ± 87.08
49.76	32.20 ± 17.63	24.68 ± 9.26	24.92 ± 9.27	194.00 ± 82.66

S5.2 Dihydrogen Activation

Table S8: Mean values of the numbers of samples for each of the target values for the dihydrogen activation dataset with the random and two local search algorithms, averaged over 25 repeats. Metrics averaged over these values are reported in the main manuscript.

Barrier	Random Search	Local Search	Guided Local
1.6	957.44 ± 687.61	208.68 ± 197.04	180.12 ± 142.69
3.6	95.96 ± 78.87	50.84 ± 31.95	78.08 ± 71.85
5.6	24.2 ± 20.99	31.8 ± 27.72	31.24 ± 26.78
7.6	25.36 ± 34.57	21.12 ± 18.83	21.68 ± 14.48
9.6	12.28 ± 12.1	20.08 ± 19.23	16.52 ± 11.77
11.6	9.08 ± 9.28	18.68 ± 14.26	19.08 ± 12.5
13.6	16.64 ± 20.1	17.12 ± 16.35	21.8 ± 18.15
15.6	18.56 ± 18.46	24.2 ± 19.67	23.16 ± 15.53
17.6	28.44 ± 24.29	31.8 ± 25.56	37.52 ± 27.01
19.6	37.2 ± 27.17	38.12 ± 23.8	26.76 ± 14.02
21.6	150.76 ± 152.51	63.32 ± 31.48	39.36 ± 16.37
23.6	315.2 ± 333.14	138.92 ± 57.63	57.52 ± 22.62
25.6	2455.12 ± 1351.6	1062.4 ± 832.55	2817.28 ± 1132.88

Table S9: Mean values of the numbers of samples for each of the target values for the dihydrogen activation dataset with the ML, Bayesian and genetic algorithms, averaged over 25 repeats. Metrics averaged over these values are reported in the main manuscript.

Barrier	ML (no barr.)	ML Search	Bayes. Opt.	Genetic Alg.
1.6	80.60 ± 67.83	14.72 ± 8.06	17.92 ± 15.44	202.16 ± 140.05
3.6	21.84 ± 30.90	9.52 ± 2.10	10.08 ± 3.25	38.56 ± 38.17
5.6	12.40 ± 5.31	9.44 ± 2.95	9.48 ± 2.94	16.92 ± 15.21
7.6	9.88 ± 3.23	10.04 ± 3.01	12.12 ± 6.13	9.60 ± 11.02
9.6	8.76 ± 2.16	8.76 ± 2.55	9.20 ± 3.20	12.52 ± 10.61
11.6	10.12 ± 5.01	8.76 ± 2.32	9.12 ± 2.73	7.72 ± 6.86
13.6	11.04 ± 5.94	8.24 ± 1.84	8.92 ± 2.30	9.20 ± 8.43
15.6	9.44 ± 2.74	8.80 ± 2.45	9.72 ± 2.85	12.88 ± 10.37
17.6	14.16 ± 8.50	9.88 ± 2.76	9.88 ± 2.80	18.20 ± 15.18
19.6	14.60 ± 7.41	9.48 ± 2.10	9.32 ± 2.20	24.36 ± 20.00
21.6	22.04 ± 17.56	10.64 ± 3.57	10.32 ± 2.65	65.20 ± 61.54
23.6	43.76 ± 28.71	7.76 ± 1.34	7.80 ± 1.36	160.28 ± 137.68
25.6	249.48 ± 175.09	9.96 ± 3.35	9.96 ± 3.67	1882.28 ± 577.27

S5.3 S_N2

Table S10: Mean values of the numbers of samples for each of the target values for the S_N2 dataset with the random and two local search algorithms, averaged over 25 repeats. Metrics averaged over these values are reported in the main manuscript.

Barrier	Random Search	Local Search	Guided Local
-42.9	3111.72 ± 1745.34	2438.28 ± 1890.58	4430.0 ± 1969.72
-15.71	4187.04 ± 2222.52	1895.04 ± 974.02	2231.52 ± 1021.08
-14.63	4532.84 ± 1889.3	1934.24 ± 1452.95	2010.96 ± 1350.86
-13.29	3819.28 ± 1978.32	3946.32 ± 1862.49	840.44 ± 702.09
-10.61	1718.88 ± 1228.18	576.04 ± 500.17	964.76 ± 758.59
-9.15	1789.16 ± 1325.42	618.44 ± 450.94	629.16 ± 472.93
-6.58	1763.2 ± 1117.16	437.44 ± 371.82	320.36 ± 261.87
-4.93	805.48 ± 672.01	168.48 ± 214.36	133.4 ± 85.04
-2.92	322.56 ± 295.74	127.44 ± 109.61	134.8 ± 97.41
-0.87	228.72 ± 204.74	110.24 ± 101.46	99.2 ± 92.87
1.07	197.36 ± 193.24	91.12 ± 76.22	79.12 ± 56.37
3.11	111.96 ± 99.79	64.04 ± 50.31	71.16 ± 57.89
5.09	106.2 ± 108.45	57.2 ± 47.88	54.72 ± 37.08
7.11	72.16 ± 65.82	56.08 ± 29.08	55.68 ± 42.83
9.09	59.6 ± 62.25	64.24 ± 50.28	59.08 ± 38.36
11.1	64.44 ± 58.48	51.88 ± 36.32	63.64 ± 63.63
13.14	61.36 ± 55.2	43.92 ± 31.62	33.72 ± 26.37
15.1	34.08 ± 35.52	39.68 ± 27.24	38.64 ± 25.85
17.11	64.64 ± 82.08	42.92 ± 27.46	42.76 ± 31.5
19.1	37.68 ± 33.13	38.28 ± 22.91	43.76 ± 28.06
21.11	47.76 ± 38.42	36.08 ± 29.28	34.56 ± 21.44
23.11	65.12 ± 52.23	46.48 ± 35.84	61.2 ± 58.2
25.13	48.36 ± 33.96	51.04 ± 34.81	35.16 ± 20.89
27.11	58.96 ± 58.79	48.92 ± 31.58	40.68 ± 27.1
29.11	79.44 ± 89.63	53.0 ± 42.62	55.64 ± 54.2
31.06	95.04 ± 88.95	48.88 ± 28.82	42.84 ± 20.66
33.17	93.2 ± 81.46	81.6 ± 61.3	53.56 ± 44.78
35.13	110.84 ± 86.12	70.0 ± 44.35	70.12 ± 55.33
37.11	163.8 ± 169.11	109.92 ± 116.84	72.48 ± 68.03
39.05	126.72 ± 138.16	75.52 ± 47.62	73.72 ± 63.1
41.06	120.68 ± 105.43	88.48 ± 65.48	57.32 ± 48.37
43.15	144.76 ± 121.09	86.32 ± 57.23	58.6 ± 57.72
45.09	154.04 ± 126.08	82.0 ± 39.63	36.24 ± 15.76
47.1	172.32 ± 125.61	93.84 ± 62.01	58.32 ± 42.54
49.08	157.56 ± 132.27	106.52 ± 74.15	66.08 ± 51.68
51.1	232.52 ± 194.58	111.04 ± 77.04	57.2 ± 45.73
53.13	653.16 ± 607.22	214.0 ± 159.5	82.08 ± 57.63
55.06	558.16 ± 587.08	195.12 ± 136.24	81.28 ± 58.22
57.02	1691.24 ± 1417.0	330.24 ± 268.37	107.6 ± 77.3
60.0	3218.68 ± 2014.03	2502.28 ± 1215.12	4421.16 ± 1918.62
61.01	3669.96 ± 2257.0	412.64 ± 321.05	118.28 ± 77.92
62.4	4026.76 ± 2002.59	2096.72 ± 1480.81	865.28 ± 637.25

Table S11: Mean values of the numbers of samples for each of the target values for the S_N2 dataset with the ML, Bayesian and genetic algorithms, averaged over 25 repeats. Metrics averaged over these values are reported in the main manuscript.

Barrier	ML (no barr.)	ML Search	Bayes. Opt.	Genetic Alg.
-42.9	617.80 ± 168.25	13.44 ± 3.71	13.36 ± 3.60	999.88 ± 567.79
-15.71	468.60 ± 332.73	16.56 ± 4.64	19.44 ± 8.45	1032.88 ± 733.74
-14.63	517.76 ± 262.05	16.00 ± 4.68	18.24 ± 4.16	682.92 ± 423.06
-13.29	1399.76 ± 34.75	87.28 ± 70.47	77.88 ± 59.23	2379.84 ± 938.43
-10.61	207.44 ± 146.30	16.20 ± 4.74	17.04 ± 5.01	496.80 ± 392.40
-9.15	264.08 ± 240.63	18.40 ± 5.37	23.36 ± 11.61	721.04 ± 498.44
-6.58	172.24 ± 88.25	28.20 ± 12.96	36.56 ± 22.52	705.68 ± 421.80
-4.93	81.84 ± 72.43	29.64 ± 21.47	30.92 ± 25.19	207.68 ± 142.00
-2.92	78.12 ± 64.22	31.16 ± 13.60	38.80 ± 28.56	135.88 ± 97.86
-0.87	50.36 ± 43.50	33.36 ± 22.88	31.16 ± 16.99	99.84 ± 80.18
1.07	38.52 ± 24.98	31.00 ± 13.40	44.40 ± 35.72	75.28 ± 58.02
3.11	40.16 ± 24.28	26.88 ± 17.08	31.20 ± 20.70	82.44 ± 66.60
5.09	33.84 ± 18.31	33.20 ± 20.15	38.88 ± 17.86	102.84 ± 98.14
7.11	33.36 ± 24.53	23.68 ± 13.45	22.60 ± 10.93	64.68 ± 46.29
9.09	39.28 ± 37.05	22.28 ± 11.14	29.32 ± 21.84	69.40 ± 56.22
11.1	34.64 ± 24.08	25.40 ± 14.19	26.28 ± 11.24	60.20 ± 59.23
13.14	30.64 ± 19.65	22.64 ± 10.73	24.36 ± 14.75	80.96 ± 69.97
15.1	26.48 ± 18.66	18.20 ± 5.41	28.36 ± 17.78	33.32 ± 33.85
17.11	30.84 ± 21.77	19.60 ± 9.37	27.96 ± 17.50	31.20 ± 40.59
19.1	29.72 ± 16.23	23.56 ± 9.20	29.52 ± 15.59	52.28 ± 72.02
21.11	32.28 ± 17.90	28.36 ± 15.14	25.04 ± 12.29	33.00 ± 33.34
23.11	35.16 ± 25.97	24.60 ± 12.52	27.20 ± 19.25	50.96 ± 49.60
25.13	33.76 ± 23.63	22.28 ± 8.62	32.44 ± 26.15	34.84 ± 29.44
27.11	30.36 ± 19.50	22.84 ± 11.08	30.08 ± 24.03	32.92 ± 24.71
29.11	35.28 ± 25.94	31.04 ± 19.57	36.36 ± 25.28	55.64 ± 38.19
31.06	43.04 ± 29.41	31.92 ± 16.08	39.20 ± 23.68	53.72 ± 45.76
33.17	42.72 ± 31.19	28.08 ± 13.34	40.36 ± 29.24	81.24 ± 79.17
35.13	41.96 ± 25.23	27.00 ± 15.78	30.24 ± 16.97	68.84 ± 62.87
37.11	39.80 ± 30.41	30.24 ± 18.50	36.40 ± 26.57	111.72 ± 77.21
39.05	40.44 ± 30.11	30.04 ± 16.65	33.96 ± 22.51	113.20 ± 87.77
41.06	30.64 ± 23.66	21.40 ± 11.38	23.56 ± 13.43	89.28 ± 74.40
43.15	35.08 ± 20.04	22.24 ± 12.56	37.16 ± 30.75	97.68 ± 67.96
45.09	46.00 ± 25.01	26.40 ± 12.66	34.96 ± 16.74	120.24 ± 124.51
47.1	44.68 ± 51.63	27.24 ± 11.23	25.04 ± 9.70	151.12 ± 153.03
49.08	72.00 ± 129.74	26.00 ± 12.71	29.04 ± 19.37	157.28 ± 175.02
51.1	38.88 ± 23.36	32.16 ± 15.74	26.40 ± 10.00	184.92 ± 106.96
53.13	58.48 ± 34.47	26.88 ± 13.32	26.20 ± 16.64	154.40 ± 97.80
55.06	61.84 ± 37.74	28.16 ± 14.83	26.40 ± 11.58	365.40 ± 302.68
57.02	180.72 ± 104.75	54.08 ± 26.73	60.76 ± 27.53	2197.28 ± 864.61
60.0	164.84 ± 102.18	37.20 ± 18.43	39.04 ± 20.38	771.48 ± 490.15
61.01	160.08 ± 118.54	38.20 ± 22.80	36.68 ± 24.72	1149.04 ± 721.75
62.4	189.00 ± 72.16	29.40 ± 13.77	25.36 ± 11.59	1503.60 ± 833.36

S5.4 E2

Table S12: Mean values of the numbers of samples for each of the target values for the E2 dataset with the random and two local search algorithms, averaged over 25 repeats. Metrics averaged over these values are reported in the main manuscript.

Barrier	Random Search	Local Search	Guided Local
-20.67	3875.12 ± 1913.81	1328.92 ± 1091.29	635.84 ± 489.02
-17.89	4103.96 ± 2350.31	4557.76 ± 2307.89	5492.8 ± 1687.43
-15.6	2844.24 ± 2225.9	1277.16 ± 896.34	1335.76 ± 997.86
-14.95	1272.36 ± 1358.77	442.96 ± 434.61	425.88 ± 407.36
-12.91	1477.32 ± 1268.08	478.56 ± 492.86	267.6 ± 237.06
-10.56	1154.12 ± 739.54	421.88 ± 353.97	365.4 ± 253.32
-8.63	224.36 ± 228.86	142.36 ± 106.62	119.88 ± 60.99
-6.68	127.88 ± 127.61	74.4 ± 52.3	91.32 ± 66.64
-4.69	67.04 ± 67.93	51.28 ± 35.33	60.96 ± 35.79
-2.67	33.96 ± 40.84	38.32 ± 32.08	55.28 ± 32.29
-0.68	48.08 ± 37.51	45.08 ± 31.34	68.24 ± 38.81
1.32	56.48 ± 46.47	38.64 ± 35.39	59.68 ± 41.38
3.34	51.68 ± 44.01	63.84 ± 37.0	68.84 ± 34.6
5.33	80.2 ± 70.18	56.88 ± 39.69	85.96 ± 63.17
7.31	105.92 ± 86.55	93.24 ± 75.23	97.08 ± 61.83
9.35	108.32 ± 94.09	70.48 ± 47.3	77.52 ± 56.76
11.21	176.56 ± 155.56	95.4 ± 62.29	99.6 ± 52.76
13.35	171.2 ± 164.63	93.12 ± 55.47	96.84 ± 53.09
15.33	231.88 ± 231.91	109.64 ± 77.51	117.16 ± 73.03
17.31	287.6 ± 227.4	141.92 ± 98.68	177.24 ± 129.61
19.29	205.84 ± 216.39	141.08 ± 106.24	226.72 ± 163.95
21.44	226.36 ± 222.2	159.32 ± 117.57	280.52 ± 193.3
23.33	269.04 ± 191.86	148.56 ± 94.6	277.04 ± 271.76
25.27	156.28 ± 133.16	132.68 ± 128.61	201.04 ± 168.87
27.31	239.96 ± 268.13	122.76 ± 102.66	166.44 ± 132.91
29.33	283.04 ± 408.25	124.64 ± 119.33	177.4 ± 132.52
31.33	254.88 ± 254.11	129.08 ± 106.8	185.24 ± 150.32
33.32	303.08 ± 262.73	125.32 ± 100.19	239.08 ± 166.56
35.11	834.56 ± 667.18	199.48 ± 160.77	353.64 ± 232.77
37.24	972.84 ± 871.37	179.16 ± 138.04	360.04 ± 242.1
40.41	3512.24 ± 2179.1	1052.88 ± 811.61	2760.88 ± 1537.98
60.09	3181.72 ± 2059.1	2244.52 ± 938.05	3277.04 ± 1783.61
60.61	3181.72 ± 2059.1	2244.52 ± 938.05	3277.04 ± 1783.61

Table S13: Mean values of the numbers of samples for each of the target values for the E2 dataset with the ML, Bayesian and genetic algorithms, averaged over 25 repeats. Metrics averaged over these values are reported in the main manuscript.

Barrier	ML (no barr.)	ML Search	Bayes. Opt.	Genetic Alg.
-20.67	1680.48 ± 344.50	274.24 ± 262.10	173.04 ± 169.70	2323.24 ± 801.61
-17.89	890.04 ± 331.07	49.68 ± 41.59	56.72 ± 44.62	1198.04 ± 720.82
-15.6	679.36 ± 361.71	50.52 ± 32.85	57.92 ± 34.89	1527.72 ± 640.14
-14.95	252.76 ± 306.50	37.16 ± 21.45	50.88 ± 46.58	884.92 ± 543.43
-12.91	268.96 ± 258.84	96.16 ± 42.95	93.80 ± 35.71	459.28 ± 335.33
-10.56	131.76 ± 96.10	59.52 ± 41.76	61.00 ± 37.13	416.88 ± 440.54
-8.63	61.60 ± 35.69	58.84 ± 24.37	67.64 ± 38.51	171.36 ± 123.93
-6.68	71.04 ± 52.13	60.60 ± 44.19	54.60 ± 30.35	134.68 ± 168.16
-4.69	41.68 ± 41.98	32.44 ± 16.37	32.24 ± 17.28	51.68 ± 53.77
-2.67	35.28 ± 19.74	35.04 ± 16.79	30.12 ± 10.63	38.88 ± 42.24
-0.68	31.80 ± 16.45	31.16 ± 15.40	29.24 ± 11.97	56.92 ± 51.55
1.32	28.76 ± 10.92	29.96 ± 10.90	36.44 ± 19.16	65.96 ± 78.60
3.34	59.68 ± 47.04	28.28 ± 12.81	29.84 ± 13.66	131.88 ± 104.50
5.33	45.24 ± 32.93	28.28 ± 11.58	35.08 ± 18.92	132.40 ± 103.42
7.31	64.68 ± 38.32	34.24 ± 14.81	37.84 ± 16.64	177.84 ± 172.42
9.35	100.60 ± 77.56	41.40 ± 27.67	43.68 ± 23.06	134.52 ± 155.93
11.21	106.68 ± 98.84	34.24 ± 11.78	39.84 ± 22.01	229.24 ± 245.18
13.35	135.04 ± 93.18	41.60 ± 19.66	59.68 ± 36.09	309.80 ± 275.43
15.33	99.92 ± 43.44	82.32 ± 45.73	77.32 ± 56.28	224.20 ± 209.67
17.31	93.56 ± 69.92	75.76 ± 45.92	73.52 ± 46.36	174.36 ± 150.67
19.29	89.08 ± 56.92	61.08 ± 49.87	75.20 ± 50.38	112.12 ± 97.73
21.44	142.00 ± 145.73	88.68 ± 61.87	107.52 ± 75.53	143.00 ± 130.82
23.33	145.12 ± 204.01	98.40 ± 55.79	108.96 ± 83.82	182.04 ± 226.79
25.27	129.36 ± 170.16	78.08 ± 51.23	119.44 ± 119.31	189.92 ± 186.35
27.31	139.72 ± 167.75	48.20 ± 32.84	98.92 ± 78.51	127.16 ± 117.63
29.33	107.12 ± 165.53	51.84 ± 25.32	87.92 ± 79.99	132.96 ± 164.78
31.33	111.04 ± 137.74	65.56 ± 65.51	74.32 ± 45.54	159.16 ± 156.93
33.32	167.76 ± 194.06	86.76 ± 68.36	98.12 ± 86.25	196.08 ± 157.94
35.11	181.36 ± 170.65	221.24 ± 150.72	263.20 ± 151.88	1061.32 ± 671.58
37.24	180.80 ± 172.14	303.84 ± 148.89	284.56 ± 185.76	920.00 ± 795.45
40.41	488.88 ± 296.64	248.80 ± 157.63	276.52 ± 184.18	2375.52 ± 1016.40
60.09	270.40 ± 192.90	23.28 ± 8.92	22.88 ± 8.73	2300.28 ± 1245.76
60.61	262.84 ± 142.83	20.72 ± 9.20	22.20 ± 11.42	2300.28 ± 1245.76

S6 ML Model Analysis

S6.1 Feature Importances

Figures S5 to S14 show the relative values of the feature importances of the ML model in our main search algorithm as the numbers of samples in the training sets increases. Feature importances are calculated with the permutation importance method as implemented in *scikit-learn*.[12] We ran these experiments on the aza-Michael addition and dihydrogen activation dataset for target barrier values at the minimum, maximum, middle points in the barrier distributions, as well as points in between (this gives us targets values of 12.2, 15.4, 31.0, 45.9 and 49.8 kcal mol^{-1} for the aza-Michael addition and 1.6, 6.8, 13.6, 20.4 and 25.6 for the dihydrogen activation). We repeat each experiment with a different barrier target 25 times and report the mean values of the feature importances in Figures S5 to S14. We note that since each sampling run can terminate after a different number of samples each time, the numbers of search runs that make up the average feature importances in the Figures decreases with the sampling number. In particular, for the aza-Michael addition results, the importances from approximately 30 samples through to 60 are mostly due to single unlucky runs, and therefore, the interpretation of the importances beyond approximately 30 samples should be treated with caution. We note also that due to our search procedure, the training and test sets are growing and shrinking respectively with each sampling iteration. Therefore, the feature importances from one iteration to the next are not truly comparable with each other since they come from models trained on slightly different datasets. However, these values should still represent the approximate behaviour of the model as the sample numbers increase.

As may be seen from these Figures, the low level approximate barriers are clearly the most important features by a significant margin, particularly in the dihydrogen activation dataset. For each of the target values, we also see lower importances for each of the one-hot encoded bits, however, we do not see any consistent patterns in these values between barrier

targets. Thus, it seems that the model primarily functions through the use of the low-level barriers as its primary source of information with corrections from the one-hot encoded R-group features that are most likely being constantly updated as the algorithm samples more data (hence the lack of consistent pattern across any of the target barriers as the sampling proceeds).

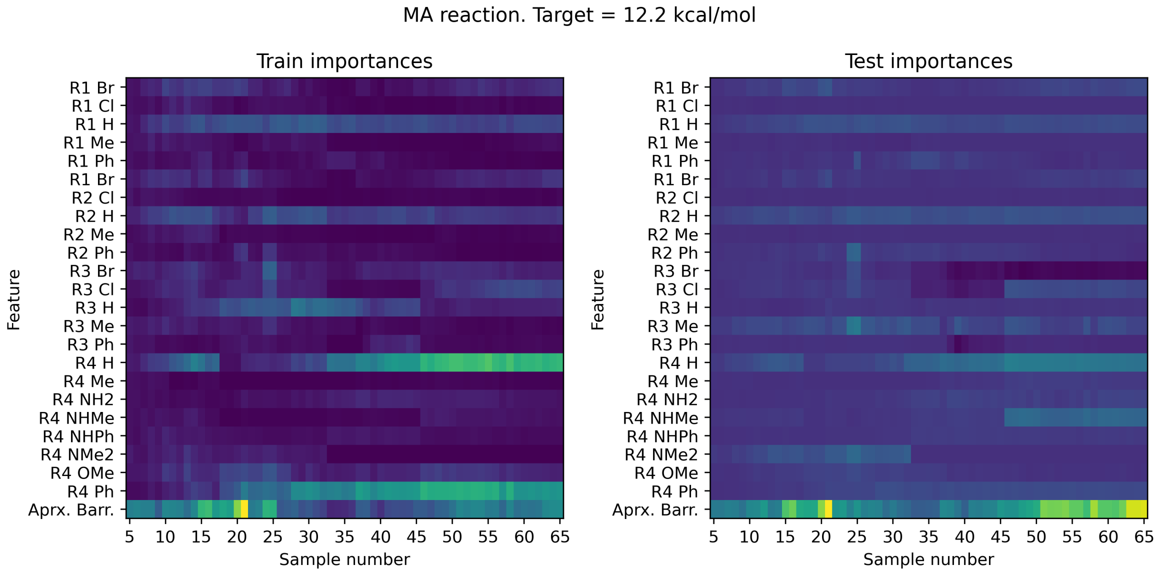

Figure S5: Permutation feature importances of the ML model in our main search algorithm for the training and test sets as the number of samples in the training set increases for the aza-Michael addition dataset with a barrier target of 12.2 kcal mol^{-1}. Yellow and green points correspond to higher relative feature importances and purple and blue correspond to lower relative feature importances.

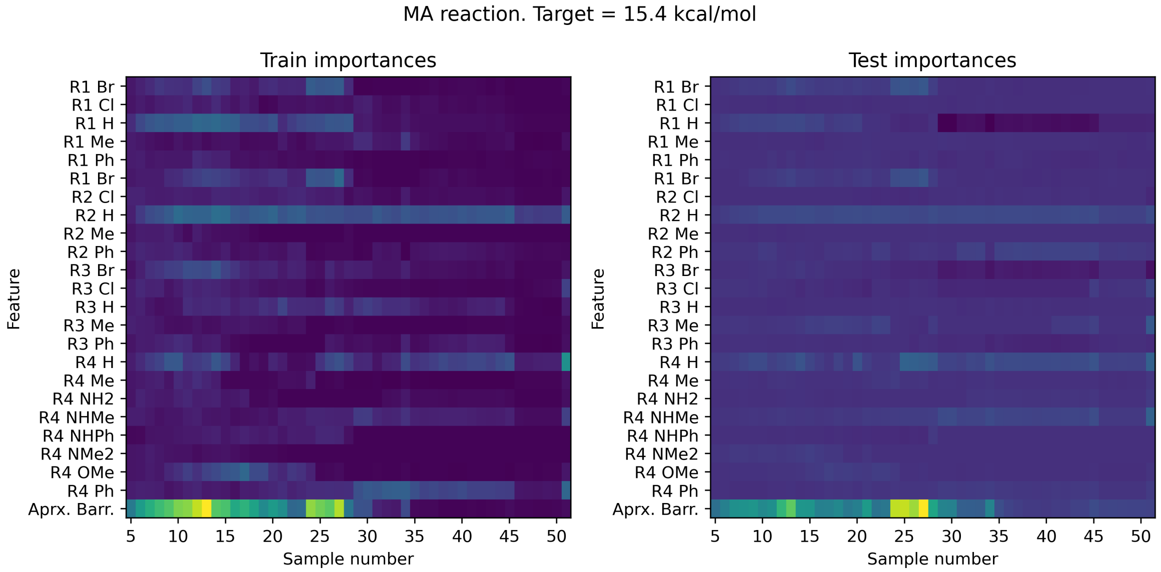

Figure S6: Permutation feature importances of the ML model in our main search algorithm for the training and test sets as the number of samples in the training set increases for the aza-Michael addition dataset with a barrier target of 15.4 kcal mol^{-1}. Yellow and green points correspond to higher relative feature importances and purple and blue correspond to lower relative feature importances.

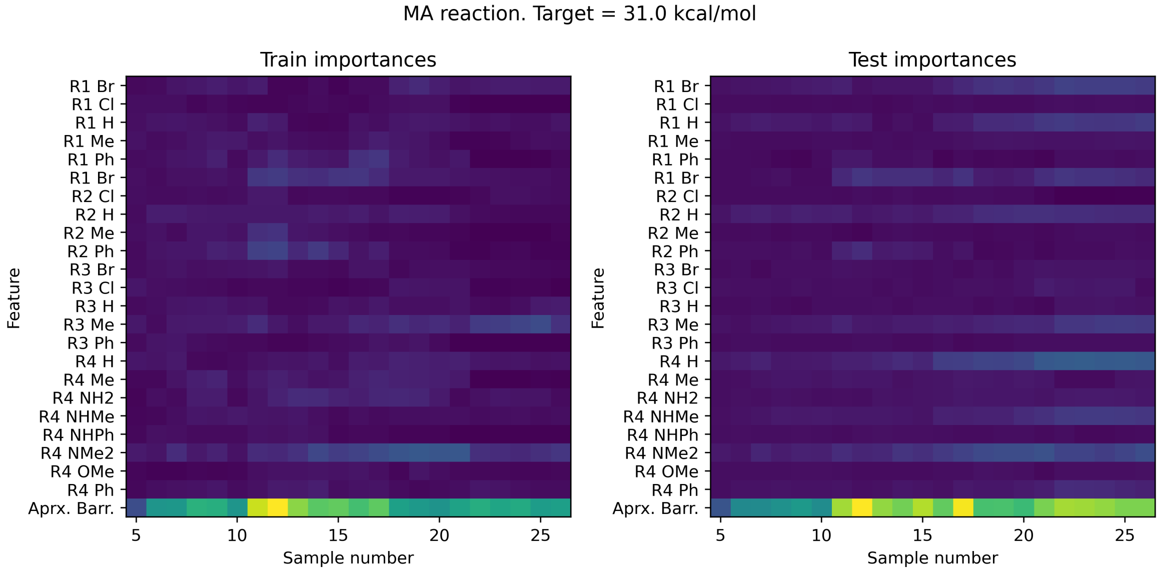

Figure S7: Permutation feature importances of the ML model in our main search algorithm for the training and test sets as the number of samples in the training set increases for the aza-Michael addition dataset with a barrier target of 31.0 kcal mol^{-1}. Yellow and green points correspond to higher relative feature importances and purple and blue correspond to lower relative feature importances.

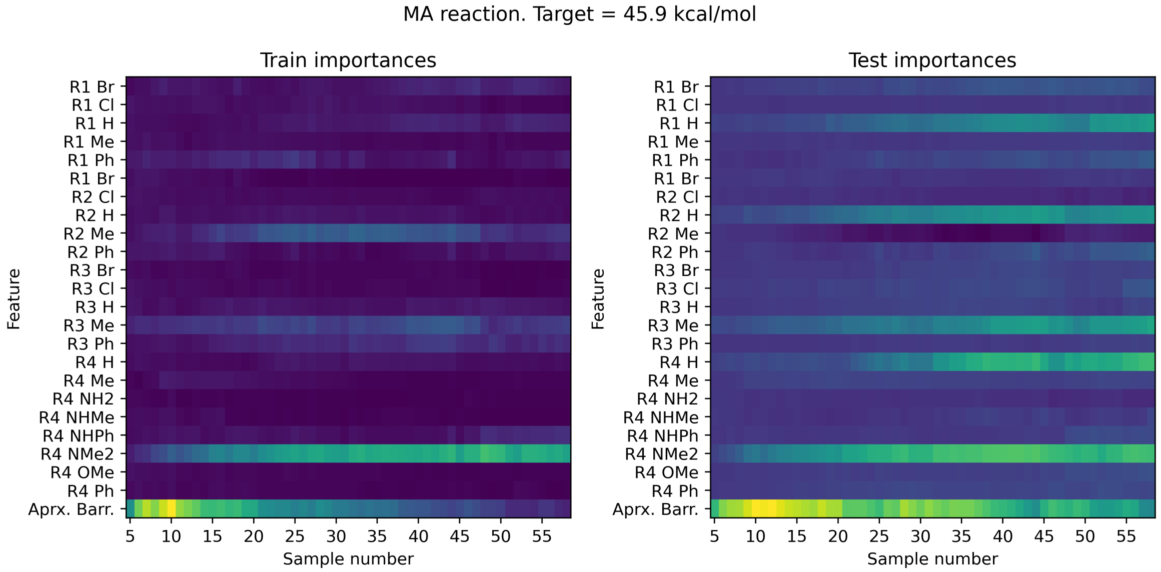

Figure S8: Permutation feature importances of the ML model in our main search algorithm for the training and test sets as the number of samples in the training set increases for the aza-Michael addition dataset with a barrier target of 45.9 kcal mol^{-1}. Yellow and green points correspond to higher relative feature importances and purple and blue correspond to lower relative feature importances.

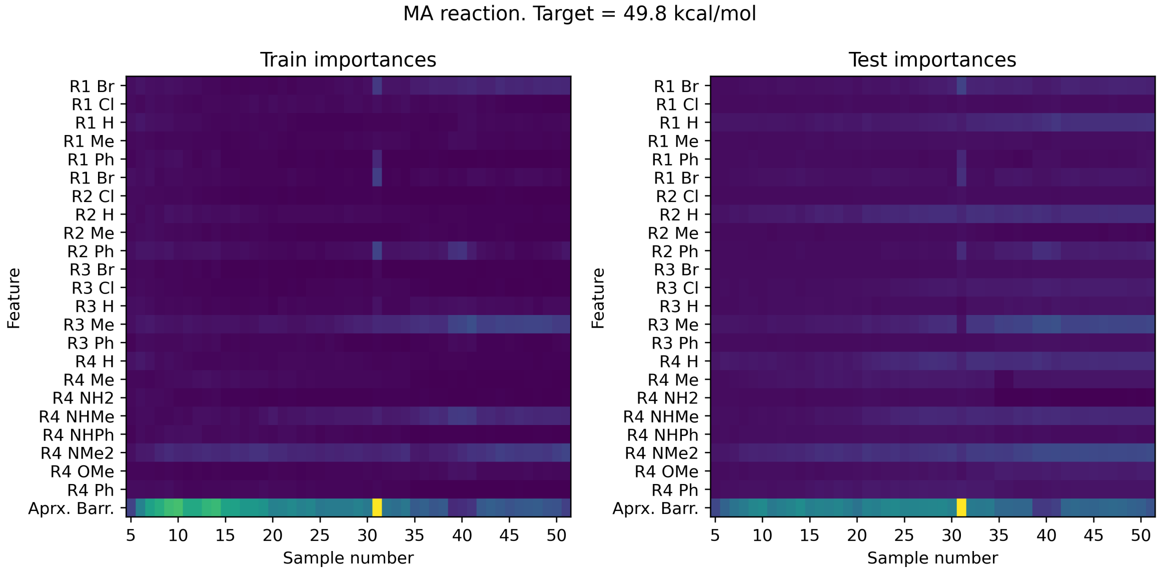

Figure S9: Permutation feature importances of the ML model in our main search algorithm for the training and test sets as the number of samples in the training set increases for the aza-Michael addition dataset with a barrier target of 49.8 kcal mol^{-1}. Yellow and green points correspond to higher relative feature importances and purple and blue correspond to lower relative feature importances.

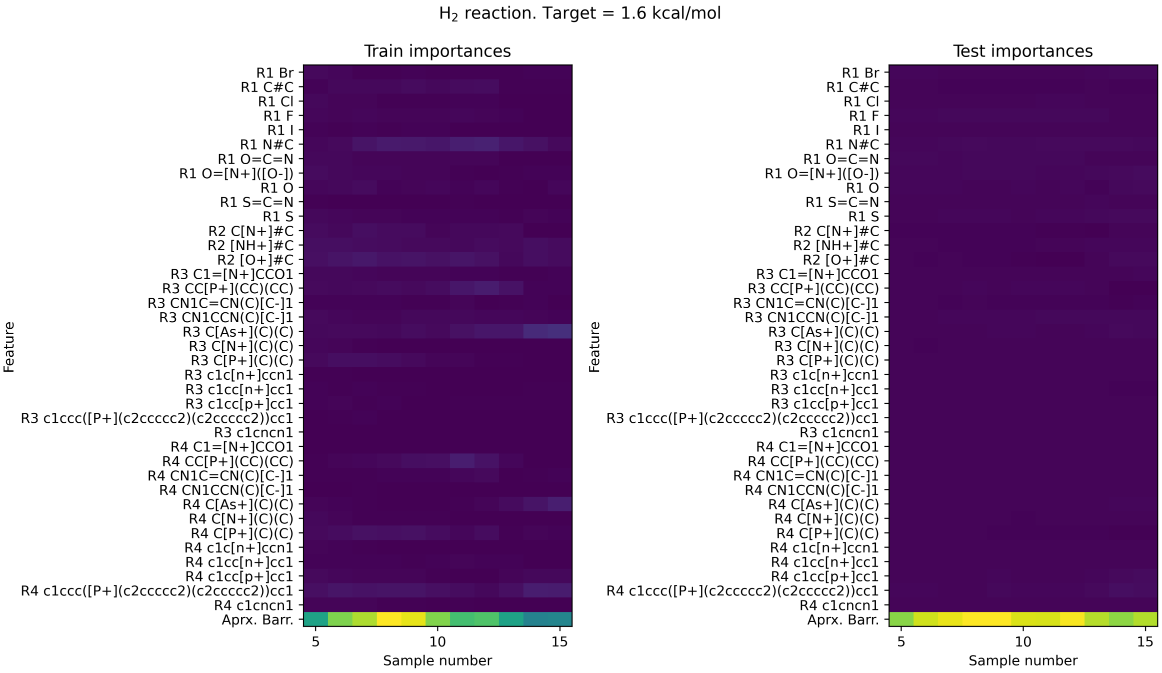

Figure S10: Permutation feature importances of the ML model in our main search algorithm for the training and test sets as the number of samples in the training set increases for the dihydrogen activation dataset with a barrier target of 1.6 kcal mol^{-1}. Yellow and green points correspond to higher relative feature importances and purple and blue correspond to lower relative feature importances.

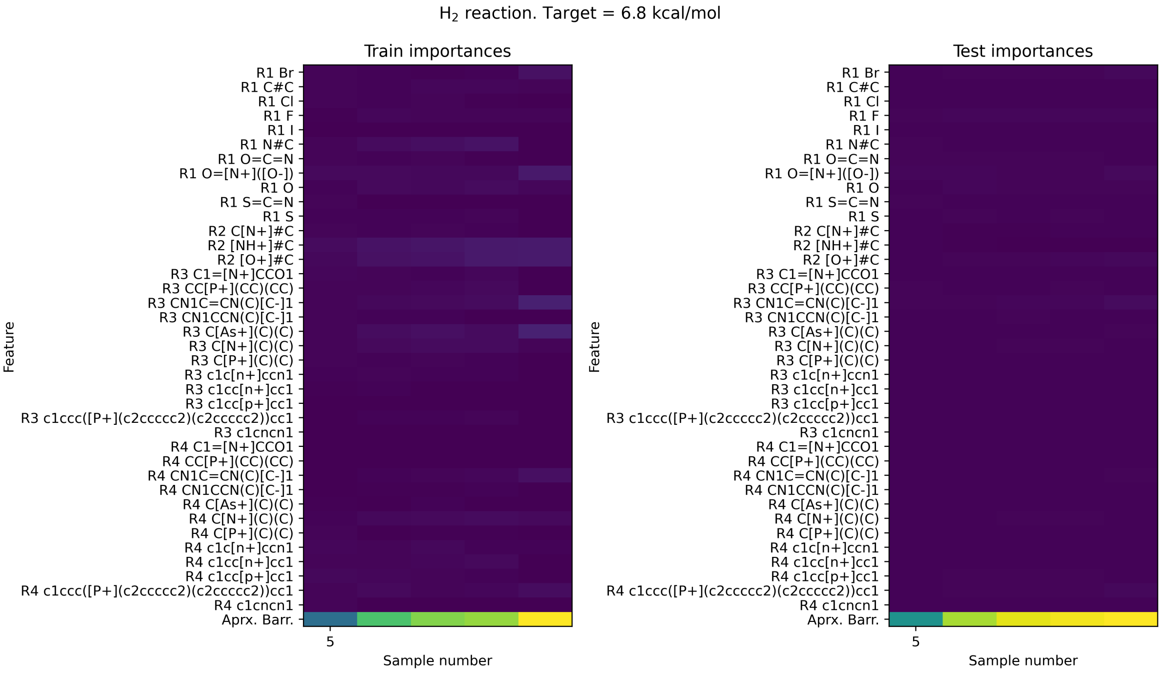

Figure S11: Permutation feature importances of the ML model in our main search algorithm for the training and test sets as the number of samples in the training set increases for the dihydrogen activation dataset with a barrier target of 6.8 kcal mol^{-1}. Yellow and green points correspond to higher relative feature importances and purple and blue correspond to lower relative feature importances.

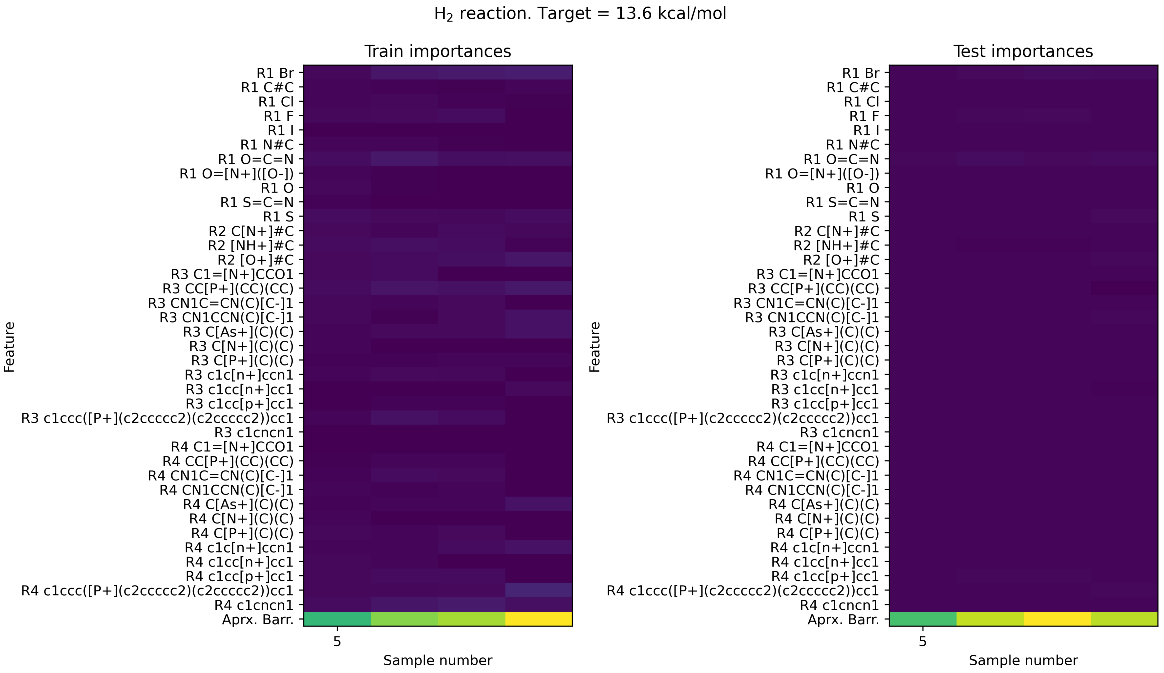

Figure S12: Permutation feature importances of the ML model in our main search algorithm for the training and test sets as the number of samples in the training set increases for the dihydrogen activation dataset with a barrier target of 13.6 kcal mol^{-1}. Yellow and green points correspond to higher relative feature importances and purple and blue correspond to lower relative feature importances.

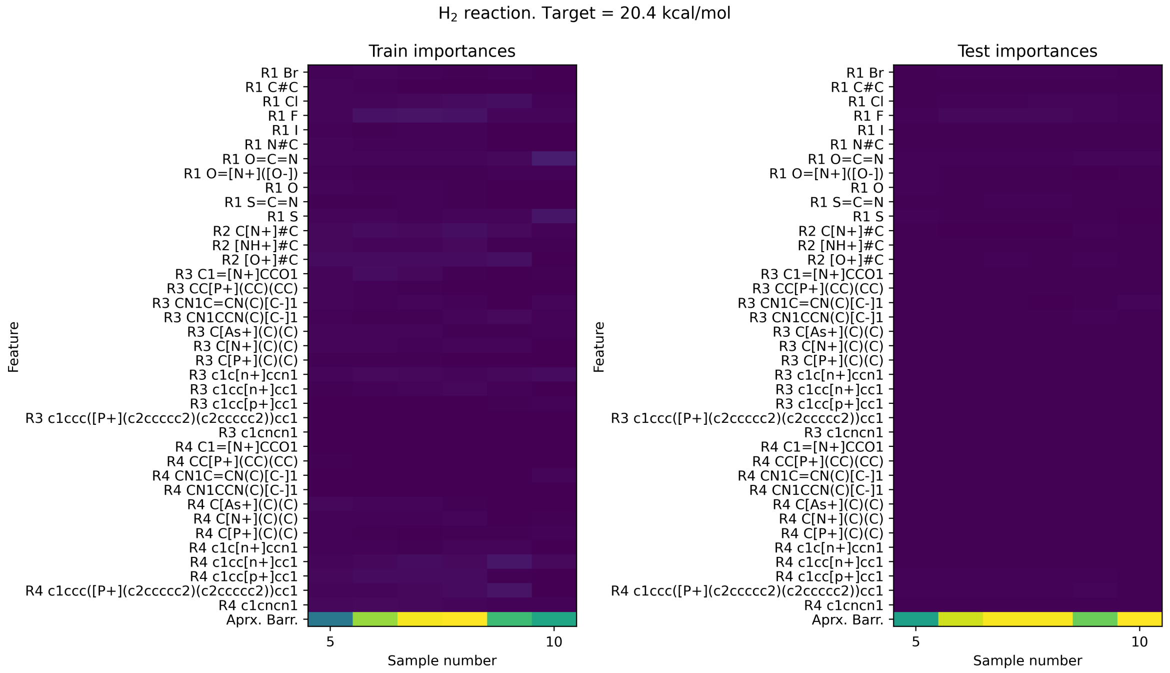

Figure S13: Permutation feature importances of the ML model in our main search algorithm for the training and test sets as the number of samples in the training set increases for the dihydrogen activation dataset with a barrier target of 20.4 kcal mol^{-1}. Yellow and green points correspond to higher relative feature importances and purple and blue correspond to lower relative feature importances.

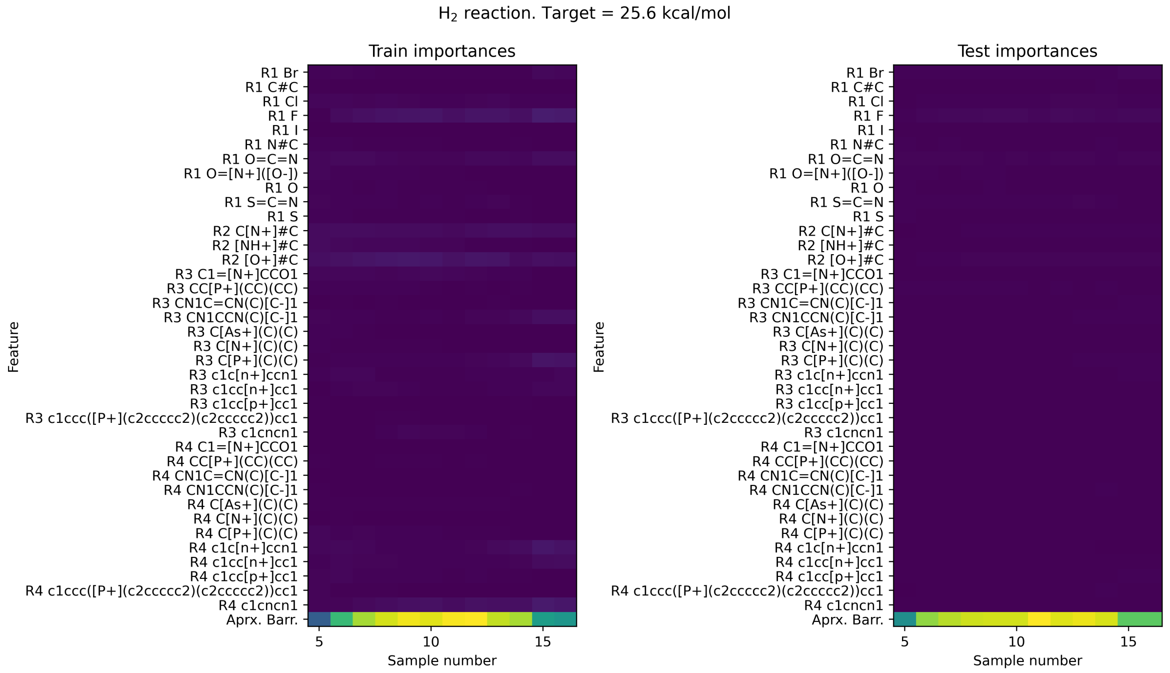

Figure S14: Permutation feature importances of the ML model in our main search algorithm for the training and test sets as the number of samples in the training set increases for the dihydrogen activation dataset with a barrier target of 25.6 kcal mol^{-1}. Yellow and green points correspond to higher relative feature importances and purple and blue correspond to lower relative feature importances.

S6.2 Model Performance

Figure S15 shows the train and test errors from the ML model in our main search algorithm as the number of samples increases. We ran these tests with the same sets of barriers targets for the aza-Michael addition and dihydrogen activation datasets as in our feature importances tests. We also ran this model performance assessment with 25 repeats and report the average values of the train and test mean absolute errors in Figure S15. We again note that the sampling sequences vary in length and that the errors at sampling numbers from approximately 30 to 60 in the aza-Michael addition results are mostly due to single unlucky runs and therefore, those errors should be analysed with less statistical certainty than errors from smaller sampling sizes. Also note that the errors from one iteration to the next are technically not truly comparable with each other, due to the fact that at each iteration the training and held-out test sets are of different sizes. However, these results should still give approximate indications of the performance of the model as the search proceeds.

Figure S15 shows the very typical trends expected from a ML model as the training set size increases: the test error shows a downward trend and the training error tends to increase as the training set becomes more varied. Interestingly, the errors of the machine learning model can be well above the chemical accuracy threshold of 1 kcal mol^{-1} and the search algorithm is still able to locate a reaction with a barrier within 1 kcal mol^{-1} of the target value.

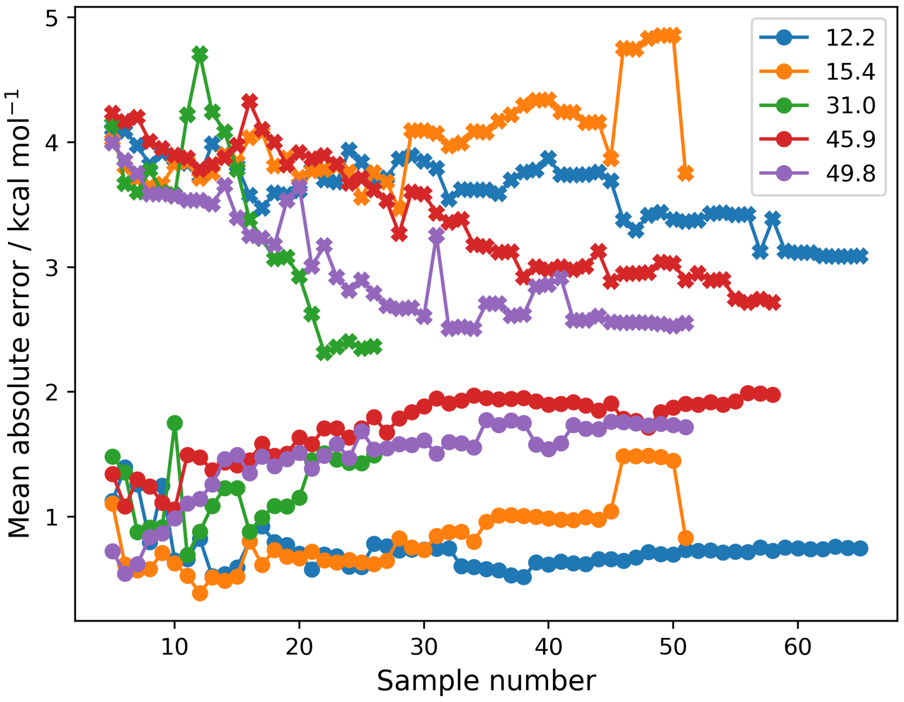

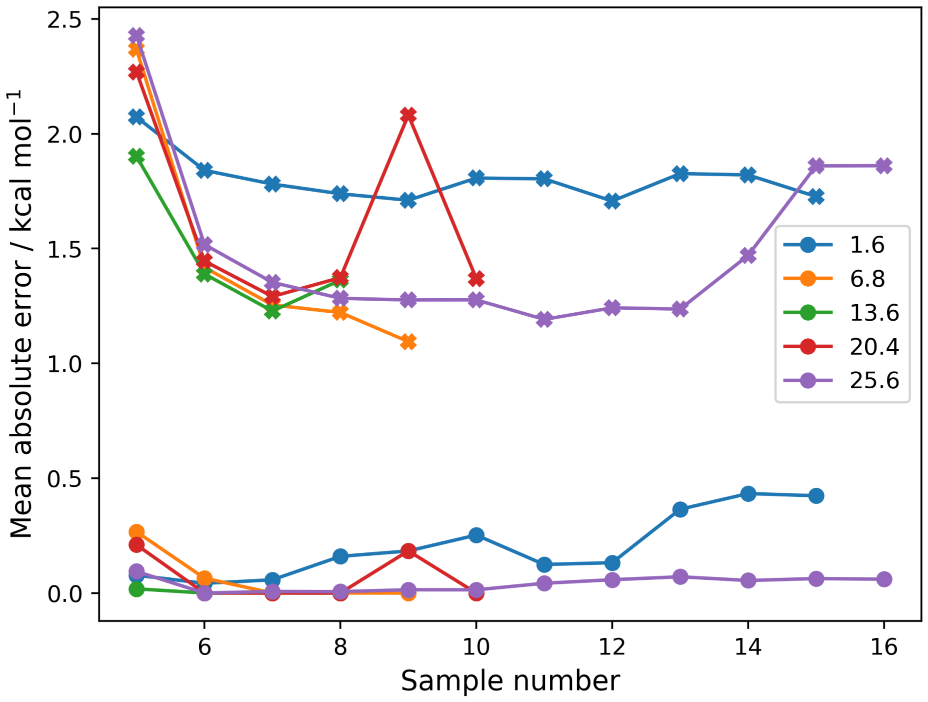

Figure S15: Average train (circles) and test (crosses) errors for the ML model in our main search algorithm applied to the aza-Michael addition (top) and dihydrogen activation (bottom) dataset for each of the barrier target values shown in the legends (in kcal mol^{-1}) as the sample number increases.

S6.3 Scrambled Features

For our final test of the ML algorithm we ran an experiment in which we randomly shuffled the one-hot encoding representations for each R-group, as well as the low-level barrier feature, between all of the reactions in the dataset in order to check that the model was learning a meaningful relationship between the features and the activation barrier, rather than just overfitting. We ran this test on the aza-Michael addition and dihydrogen activation datasets with the same target barriers as in our main results and we repeat the experiments on each barrier target 25 times. Table S14 and Table S15 show the average numbers of samples the ML algorithm required for each of the target values.

When using scrambled input features, the search algorithm performs noticeably worse than before. In particular, this is much more apparent at the very tail ends of the barrier distribution, where algorithms that are essentially random sampling perform much more poorly. Thus, it appears to be the case that a meaningful relationship between the input features and the activation barriers was being learnt by the model during the search procedure.

Table S14: The average numbers of samples required by the ML search algorithm to find a reaction within 1 kcal mol^{-1} of each of the target values for the aza-Michael addition reaction with scrambled features.

Barrier	Sample Number
12.23	493.12
13.97	242.64
16.52	141.48
18.33	30.72
20.24	14.96
22.20	11.68
24.23	10.36
26.23	9.24
28.22	8.52
30.21	6.52
32.21	10.40
34.21	16.08
36.24	19.84
38.14	51.12
40.20	74.72
42.66	217.52
43.64	267.24
45.86	480.00
47.22	437.40
49.76	476.40

Table S15: The average numbers of samples required by the ML search algorithm to find a reaction within 1 kcal mol^{-1} of each of the target values for the dihydrogen activation reaction with scrambled features.

Barrier	Sample Number
1.6	539.92
3.6	65.44
5.6	20.52
7.6	14.48
9.6	14.32
11.6	11.52
13.6	14.12
15.6	11.12
17.6	17.00
19.6	27.28
21.6	74.04
23.6	149.72
25.6	1205.76

S7 Old E2 and S_N2 Results with Scrambled Low-Level Barriers

As discussed in the methods section of the main manuscript, the E2 and S_N2 datasets contain structures duplicated by symmetry which were removed before our ML optimization. However, in the original version of this paper, a bug caused by one missing line of code meant that the low-level barriers of the symmetry-duplicated E2 and S_N2 structures were not filtered along with the reactions and high-level barriers. Therefore, the relationship between the low-level and high-level barriers for these experiments was effectively scrambled, artificially reducing the correlation between the low- and high-level barriers. After this scrambling occurred, the Pearson R^2 correlation coefficient between the low- and high-level E2 barriers was 0.56 and the value between the low- and high-level S_N2 barriers was 0.65. This highlights the usefulness of the idea of using the approximate, low-level barrier feature; even with this error and the consequent weaker correlations, we were still able to achieve the impressive results for these datasets reported previously. Therefore, here we present the old results for the E2 and S_N2 datasets that were affected by the low-level barrier mistake, as illustration of the usefulness of the approach even when the low-level data are not in an ideal condition.

Table S16: The mean numbers of sampled reactions required to obtain barriers within 1 kcal mol^{-1} of a target value, averaged over all of the target values for each dataset, that were reported in the original version of this paper using the scrambled low-level E2 and S_N2 barriers.

data set (% complete)	Random Search	Local Search	Guided Local	ML (no barr.)	ML Search	Bayes. Opt.	Genetic Alg.
S_N2 (44.1%)	923.27	470.05	449.67	134.58	**47.75**	49.48	373.64
E2 (25.6%)	912.72	503.81	653.97	221.04	**100.24**	116.33	577.08

Table S17: The mean numbers of sampled reactions required to obtain barriers within 1 kcal mol^{-1} of a target value, averaged over all except the 5 highest and 5 lowest target values, that were reported in the original version of this paper using the scrambled low-level E2 and S_N2 barriers.

data set (% complete)	Random Search	Local Search	Guided Local	ML (no barr.)	ML Search	Bayes. Opt.	Genetic Alg.
S_N2 (44.1%)	257.59	106.72	87.97	52.65	**33.41**	35.42	128.55
E2 (25.6%)	211.47	113.91	147.59	92.98	**70.37**	86.73	160.57

Table S18: The mean numbers of sampled reactions required to obtain barriers within 1 kcal mol^{-1} of a target value, averaged over the 5 highest and 5 lowest target values, that were reported in the original version of this paper using the scrambled low-level E2 and S_N2 barriers.

data set (% complete)	Random Search	Local Search	Guided Local	ML (no barr.)	ML Search	Bayes. Opt.	Genetic Alg.
S_N2 (44.1%)	3053.46	1632.69	1607.13	396.78	**93.64**	94.46	1157.91
E2 (25.6%)	2525.61	1400.59	1818.65	515.59	**168.92**	184.41	1535.06

Table S19: Mean values of the numbers of samples for each of the target values for the S_N2 dataset which used the original, scrambled low-level barriers, averaged over 25 repeats. Metrics averaged over these values are reported in Tables S16, S17 and S18.

Barrier	ML Search	Bayes. Opt.
-42.9	13.16 ± 3.53	13.20 ± 3.60
-15.71	15.92 ± 4.96	16.40 ± 6.16
-14.63	15.40 ± 3.59	17.84 ± 4.94
-13.29	246.52 ± 176.25	237.68 ± 171.64
-10.61	16.08 ± 4.58	15.96 ± 5.24
-9.15	20.88 ± 9.70	18.64 ± 6.29
-6.58	22.12 ± 7.52	27.92 ± 14.49
-4.93	20.24 ± 8.82	21.48 ± 12.96
-2.92	27.76 ± 14.34	31.12 ± 16.58
-0.87	36.52 ± 28.16	34.92 ± 22.79
1.07	31.00 ± 15.09	23.52 ± 14.83
3.11	27.52 ± 18.53	31.92 ± 28.13
5.09	36.88 ± 22.32	33.96 ± 17.31
7.11	20.44 ± 9.84	23.28 ± 9.90
9.09	28.92 ± 19.66	34.40 ± 28.28
11.1	31.56 ± 20.28	25.92 ± 14.56
13.14	28.24 ± 15.95	26.76 ± 21.56
15.1	25.04 ± 17.34	24.88 ± 17.07
17.11	31.60 ± 20.09	23.52 ± 13.96
19.1	30.52 ± 15.57	35.72 ± 19.82
21.11	31.20 ± 20.74	32.40 ± 19.95
23.11	33.48 ± 21.14	30.76 ± 22.85
25.13	34.44 ± 24.34	43.88 ± 31.20
27.11	28.68 ± 21.59	30.04 ± 21.88
29.11	35.32 ± 19.76	37.00 ± 30.95
31.06	34.76 ± 22.02	39.32 ± 24.47
33.17	47.20 ± 35.80	48.64 ± 28.55
35.13	33.68 ± 23.91	53.24 ± 46.24
37.11	44.48 ± 31.91	50.80 ± 34.80
39.05	43.76 ± 24.35	53.04 ± 26.98
41.06	39.28 ± 26.40	36.60 ± 25.90
43.15	39.32 ± 24.94	39.48 ± 30.25
45.09	66.64 ± 37.62	58.88 ± 36.64
47.1	30.60 ± 28.39	43.40 ± 32.44
49.08	38.56 ± 45.94	30.96 ± 22.79
51.1	35.12 ± 23.11	44.76 ± 32.07
53.13	33.44 ± 22.87	42.40 ± 34.51
55.06	45.32 ± 30.00	59.80 ± 36.41
57.02	169.60 ± 122.36	218.12 ± 89.14
60.0	82.32 ± 74.06	83.60 ± 71.12
61.01	177.72 ± 76.87	174.08 ± 79.88
62.4	154.32 ± 63.89	107.92 ± 40.13

Table S20: Mean values of the numbers of samples for each of the target values for the E2 dataset which used the original, scrambled low-level barriers, averaged over 25 repeats. Metrics averaged over these values are reported in Tables S16, S17 and S18.

Barrier	ML Search	Bayes. Opt.
-20.67	37.20 ± 39.93	64.88 ± 106.88
-17.89	36.24 ± 44.84	35.16 ± 40.15
-15.6	22.52 ± 7.51	23.72 ± 8.65
-14.95	27.24 ± 17.79	26.84 ± 10.09
-12.91	30.40 ± 16.31	37.72 ± 18.78
-10.56	25.68 ± 9.48	29.56 ± 17.81
-8.63	30.96 ± 13.72	37.76 ± 20.34
-6.68	33.52 ± 13.65	32.12 ± 12.92
-4.69	27.80 ± 14.08	24.16 ± 12.06
-2.67	31.88 ± 13.52	29.68 ± 11.40
-0.68	29.40 ± 15.59	27.32 ± 12.14
1.32	34.04 ± 15.24	30.84 ± 21.30
3.34	41.32 ± 28.54	56.00 ± 46.87
5.33	38.24 ± 22.92	53.84 ± 46.14
7.31	61.44 ± 40.15	88.72 ± 97.12
9.35	94.08 ± 67.97	88.36 ± 71.02
11.21	68.56 ± 42.01	80.36 ± 62.86
13.35	122.56 ± 78.60	136.96 ± 123.76
15.33	113.76 ± 76.18	159.56 ± 135.20
17.31	83.72 ± 56.89	122.36 ± 102.78
19.29	136.04 ± 115.52	194.80 ± 153.16
21.44	124.36 ± 130.14	172.80 ± 187.71
23.33	100.60 ± 126.25	106.88 ± 130.87
25.27	100.00 ± 97.91	126.68 ± 126.11
27.31	81.28 ± 115.69	94.08 ± 135.08
29.33	65.88 ± 66.30	83.32 ± 85.78
31.33	61.36 ± 65.24	74.92 ± 69.52
33.32	112.08 ± 133.86	143.72 ± 146.48
35.11	189.16 ± 172.12	138.44 ± 118.45
37.24	101.92 ± 105.36	150.84 ± 160.50
40.41	655.68 ± 287.47	615.68 ± 361.09
60.09	292.68 ± 176.23	419.20 ± 354.74
60.61	296.16 ± 270.86	331.64 ± 295.72

References

(1) Luchini, G.; Paton, R.; Alegre-Requena, J.; Rodríguez-Guerra, J.; Berquist, E.; Chen, J.; IFunes,; Velmiskina, J.; froessler,; Mayes, H.; Vejaykummar, S. S. S.; sibo, patonlab/GoodVibes. 2022; https://doi.org/10.5281/zenodo.6977304 [Accessed August 2023].

(2) Grimme, S. Supramolecular Binding Thermodynamics by Dispersion-Corrected Density Functional Theory. *Chem. Euro. J.* **2012**, *18*, 9955–9964.

(3) Russell, S. J. *Artificial intelligence a modern approach*; Pearson Education, Inc., 2010.

(4) Mockus, J. Application of Bayesian approach to numerical methods of global and stochastic optimization. *J. Glob. Optim.* **1994**, *4*, 347–365.

(5) Jones, D. R.; Schonlau, M.; Welch, W. J. Efficient Global Optimization of Expensive Black-Box Functions. *J. Glob. Optim.* **1998**, *13*, 455–492.

(6) Deep, K.; Singh, K. P.; Kansal, M.; Mohan, C. A real coded genetic algorithm for solving integer and mixed integer optimization problems. *Appl. Math. Comput.* **2009**, *212*, 505–518.

(7) Landrum, G. et al. RDKit: Open-source cheminformatics. 2023; https://www.rdkit.org [Accessed August 2023].

(8) Gasteiger, J.; Marsili, M. Iterative partial equalization of orbital electronegativity—a rapid access to atomic charges. *Tetrahedron* **1980**, *36*, 3219–3228.

(9) Hall, L. H.; Kier, L. B. *Reviews in Computational Chemistry*; John Wiley Sons, Ltd, 1991; pp 367–422.

(10) Wildman, S. A.; Crippen, G. M. Prediction of Physicochemical Parameters by Atomic Contributions. *J. Chem. Inf. Comp. Sci.* **1999**, *39*, 868–873.

(11) RDKit, Getting Started with the RDKit in Python: List of Available Descriptors. https://www.rdkit.org/docs/GettingStartedInPython.html#list-of-available-descriptors [Accessed August 2023].

(12) Pedregosa, F. et al. Scikit-learn: Machine Learning in Python. *J. Mach. Learn. Res.* **2011**, *12*, 2825–2830.
